# Repeated exposure of wheat to the fungal root pathogen *Bipolaris sorokiniana* modulates rhizosphere microbiome assembly and disease suppressiveness

**DOI:** 10.1186/s40793-023-00529-2

**Published:** 2023-12-05

**Authors:** Lilian S. Abreu Soares Costa, Mírian Rabelo de Faria, Josiane Barros Chiaramonte, Lucas W. Mendes, Edis Sepo, Mattias de Hollander, José Maurício Cunha Fernandes, Víctor J. Carrión, Wagner Bettiol, Tim H. Mauchline, Jos M. Raaijmakers, Rodrigo Mendes

**Affiliations:** 1 Embrapa Environment, Jaguariúna, Brazil; 2 Center for Nuclear Energy in Agriculture, University of São Paulo, Piracicaba, Brazil; 3 Department of Microbial Ecology, Netherlands Institute of Ecology (NIOO-KNAW), Wageningen, The Netherlands; 4 Embrapa Wheat, Passo Fundo, Brazil; 5 Institute of Biology, Leiden University, Leiden, The Netherlands; 6 Departamento de Microbiología, Facultad de Ciencias, Universidad de Málaga, Málaga, Spain; 7 Departamento de Microbiología y Protección de Cultivos, Instituto de Hortofruticultura Subtropical y Mediterránea “La Mayora”, Universidad de Málaga-Consejo Superior de Investigaciones Científicas, Málaga, Spain; 8 Sustainable Soils and Crops, Rothamsted Research, Harpenden, UK

**Keywords:** Soilborne pathogens, Plant disease suppression, Soil microbiome, Bacterial communities, Fungal communities

## Abstract

**Background:**

Disease suppressiveness of soils to fungal root pathogens is typically induced in the field by repeated infections of the host plant and concomitant changes in the taxonomic composition and functional traits of the rhizosphere microbiome. Here, we studied this remarkable phenomenon for *Bipolaris sorokiniana* in two wheat cultivars differing in resistance to this fungal root pathogen.

**Results:**

The results showed that repeated exposure of the susceptible wheat cultivar to the pathogen led to a significant reduction in disease severity after five successive growth cycles. Surprisingly, the resistant wheat cultivar, initially included as a control, showed the opposite pattern with an increase in disease severity after repeated pathogen exposure. Amplicon analyses revealed that the bacterial families *Chitinophagaceae*, *Anaerolineaceae* and *Nitrosomonadaceae* were associated with disease suppressiveness in the susceptible wheat cultivar; disease suppressiveness in the resistant wheat cultivar was also associated with *Chitinophagaceae* and a higher abundance of *Comamonadaceae*. Metagenome analysis led to the selection of 604 Biosynthetic Gene Clusters (BGCs), out of a total of 2,571 identified by AntiSMASH analysis, that were overrepresented when the soil entered the disease suppressive state. These BGCs are involved in the biosynthesis of terpenes, non-ribosomal peptides, polyketides, aryl polyenes and post-translationally modified peptides.

**Conclusion:**

Combining taxonomic and functional profiling we identified key changes in the rhizosphere microbiome during disease suppression. This illustrates how the host plant relies on the rhizosphere microbiome as the first line of defense to fight soil-borne pathogens. Microbial taxa and functions identified here can be used in novel strategies to control soil-borne fungal pathogens.

**Supplementary Information:**

The online version contains supplementary material available at 10.1186/s40793-023-00529-2.

## Background

Disease suppressive soils are the best examples of microbiome-mediated protection of plants against infections by soil-borne pathogens [1]. In suppressive soils, the roots of susceptible crop plants are protected from specific diseases despite the presence of a virulent pathogen. This intriguing phenomenon has been observed for various fungal root pathogens, such as *Rhizoctonia solani* [2, 3], *Fusarium oxysporum* [4, 5], *Fusarium culmorum* [6] and *Gaeumannomyces tritici* [7–9]. Specific disease suppression has been attributed to the enrichment of specific members of the root microbiome that interfere with the pathogen infection cycle leading to plant protection [3, 8, 10]. This specific disease suppression can be eliminated by selective heat treatment of the soil and can be transplanted to non-suppressive (i.e., conducive) soils [3, 11]. Although specific disease suppression is sensitive to soil management practices, it can be rapidly regained in the presence of the original host plant and the inducing pathogen [1].

For soil-borne fungal diseases, specific suppression typically develops after a severe disease outbreak [12, 13], suggesting that the microbiome requires time to react to the pathogen infection [1]. Carrión et al. [10] reported that enrichment of specific disease-suppressive microbial taxa and activation of specific functional traits not only occurs in the rhizosphere but is also observed for members of the endophytic root microbiome. Hence, disease suppressiveness of soils does not fall within the traditional disease triangle, where the success of a virulent fungal root pathogen relies on the susceptibility of the host plant and environmental conditions that are conducive to pathogen invasion but also depends on the composition and activity of the root microbiome [14].

Plant genetics is a key factor in pathogen resistance but also determines, in part, the composition and activities of the root microbiome [15–17]. For example, the genotype differences observed in old and modern wheat cultivars strongly influence root microbiome composition and interactions [18–20]. Hence, understanding the fundamental mechanisms underlying the connection between root microbiome assembly, plant genetics and pathogen suppression is essential to maximize disease control. Here, we investigated (i) the impact of the wheat genotype on the onset and dynamics of root disease caused by the fungal pathogen *Bipolaris sorokiniana* and (ii) the temporal changes in root microbiome composition and functions over five plant cultivation cycles. By integrating 16S rRNA gene and ITS amplicon sequencing with metagenomics, we investigated how repeated exposures of susceptible and resistant wheat genotypes to the fungal root pathogen *Bipolaris* affected the taxonomic and functional diversity of the rhizosphere microbiome and if/how these changes coincided with disease suppressiveness. A mechanistic understanding of the interplay between rhizosphere microbiome assembly and disease dynamics will provide a fundamental basis to identify microbial consortia and functional microbial traits as a novel strategy to control soil-borne fungal pathogens.

## Materials and methods

### Pathogen inoculum and soil bioassays

The fungus *Bipolaris sorokiniana* BS 0208 was provided by Embrapa Wheat (Passo Fundo RS, Brazil), and stored in mineral oil at 10 °C. The inoculum was prepared by growing *B. sorokiniana* on potato-dextrose-agar (PDA, pH 5.5 ± 0.2) medium for seven days at 21 ± 2 °C with 12 h photoperiod. Conidial suspensions (10^4^ conidia mL^−1^) were used as inoculum in the soil bioassays. The agricultural soil used in these bioassays was collected from a production area located in Ibirarema SP, Brazil (22° 55′ 45.36″ S, 50° 7′ 22.33″ W). This area has been under wheat cultivation in rotation with soybean for more than 10 years. The authorization for soil sampling is registered with the National System for the Management of Genetic Heritage and Associated Traditional Knowledge (SISGen) under number A7E50BB. The soil was collected at a depth of up to 20 cm, air-dried, and passed through a 2-mm-mesh sieve before use. Soil chemical analyses were performed in the Soil Fertility Laboratory at the “Luiz de Queiroz” College of Agriculture, University of São Paulo (ESALQ/USP), Piracicaba SP, Brazil, and are detailed in Additional file 1: Table S1.

### Screening for resistance against *Bipolaris sorokiniana*

We selected 13 wheat genotypes to investigate their response to root infection by *B. sorokiniana* (Additional file 1: Table S2). Of these 13 wheat genotypes, two landraces and five modern cultivars were previously studied for their rhizosphere microbiome community assembly [18]; the other six genotypes were recommended by Embrapa Wheat (Passo Fundo RS, Brazil) based on their resistance or susceptibility to *B. sorokiniana*. The screening to select resistant and susceptible wheat genotypes was performed in plastic pots (10 cm high and 13 cm diameter) filled with approximately 300 g of soil and each pot was sown with 10 wheat seeds. The experimental design included 13 wheat genotypes, two treatments (with or without *B. sorokiniana* inoculation), and four replicates, resulting in 104 independent pots. The plants were cultivated in climate chambers with a 12 h photoperiod at 21 ± 2 °C. The fungal pathogen was inoculated in the soil six days after sowing (vegetative stage: two leaves), by adding 1 mL of the conidia suspension (10^4^ conidia mL^−1^) to the base of each germinated seedling with a total of 10 mL of suspension per pot. The number of infected plants was scored four weeks after inoculation and the disease severity index (DSI) was calculated (adapted according to McMillan et al., 2014 [21]). DSI ranged from 0 (healthy plant) to 3 (severe symptoms): 0 = no symptoms, 1 = infected plants with slight dark lesion only on the cotyledon leaf, 2 = infected plants with moderate dark or red lesion on the stem, 3 = severe dark symptoms on the stem and above the first leaf (Additional file 1: Figure S1). The DSI was calculated for each pot using the following formula: (1 × percentage plants scored 1) + (2 × percentage plants scored 2) + (3 × percentage plants scored 3) divided by the total number of categories (3); maximum DSI 100%.

### Plant bioassays and repeated pathogen exposure

Out of the 13 wheat genotypes screened for resistance against *B. sorokiniana*, we selected two resistant and two susceptible genotypes to study the onset and dynamics of the disease. The four selected plant genotypes were cultivated in the agricultural soil in the presence or absence of *B. sorokiniana* inoculum for five successive growth cycles of four weeks each. The inoculum was reintroduced before each growth cycle in the treatments with the fungal pathogen. The bioassay was assembled in pots filled with approximately 250 g of soil. Ten wheat seeds were sown in each pot and watered to 20% (v/w). The experimental design included four wheat genotypes (two susceptible and two resistant), two treatments (with or without *B. sorokiniana* inoculum), five growth cycles, and six replicates. Pots without plants and the pathogen served as the bulk soil samples, bringing the total to 240 independent pots (samples) (Additional file 1: Figure S2). Plants were cultivated in a climate chamber with a 12 h photoperiod at 21 ± 2 °C. DSI was assessed four weeks after pathogen inoculation as described above (cycle period). For rhizosphere sampling, plants were removed from pots and gently shaken to remove excess soil adhered to the roots; then the root system was placed in a sterile plastic bag and vigorously shaken to detach the tightly adhering soil, i.e., the rhizosphere. The excess soil was returned to the pot for the next growth cycle. The samples were stored in 2 ml tubes, flash-frozen in liquid nitrogen and kept at − 80 °C until DNA extraction. Approximately 0.5 g of the root system was returned to each pot for the next growth cycle to simulate the monoculture system in the field, where parts of the plant root system remain in the soil. This process including plant cultivation, disease evaluation, and rhizosphere soil sampling was repeated for a total of five cycles.

### Rhizosphere DNA extraction and sequencing

Total DNA was extracted from 250 mg of rhizosphere soil using the Powersoil Dneasy DNA isolation kit (QIAGEN Laboratories), according to the manufacturer’s protocol. The DNA quality and concentration were determined by 0.8% sodium boric acid agarose gel electrophoresis and NanoDrop®ND-2000 Spectrophotometer (Thermo Scientific®, Wilmington, DE, USA). In addition, the DNA concentration (ng µl^−1^) was estimated using the QUBIT® 2.0 (Life Technologies®) fluorimeter and all samples were calibrated to a final volume of 20 µl (5 ng µl^−1^). The samples were lyophilized and shipped to Argonne National Laboratory for 16S rRNA and ITS region sequencing and Novogene Corporation Inc. for metagenome sequencing.

### Quantitative PCR for pathogen quantification

Quantitative PCR analysis (qPCR) was performed on the extracted DNA to quantify the abundance of the pathogen in the rhizosphere samples of the four wheat genotypes over the growth cycles. The amplification reactions used the specific primers for the pathogen *Bipolaris sorokiniana* CosA_F_519 5'-TCAAGCTGACCAAATCACCTTC-3’ [22] and CosA_R_248 5'-AATGTCGAGCTTGCCAAAGT-3’ [23]*.* The reactions were performed in a final volume of 20 µl containing 10 µl of SYBR Green qPCR Super Mix-UDG (Invitrogen by ThermoFisher Scientific), 1.0 µM of each primer, and 10 ng of DNA template. DNA amplification using a StepOnePlus System (Applied Biosystems, Foster City, CA, USA) was performed with an initial denaturation temperature at 95 °C for 10 min, followed by 40 cycles of 95 °C for 30 s, 55 °C for 30 s and 72 °C for 40 s. The Ct values (cycles threshold) were used as standards for determining the amount of DNA template in each sample. The gene copy number in different samples was expressed as the log of the gene copy numbers per gram of soil. Statistical differences between the soil samples were determined using a One-way analysis of variance and Scott-Knott (*P* < 0.05) on R v4.02 [24].

### 16S rRNA gene and ITS region amplicon sequencing and data processing

The V4 region of the bacterial 16S rRNA gene was amplified using the 515F (forward primer 5′-GTGCCAGCMGCCGCGGTAA-3′) and 806R (reverse primer − 5′-GGAC TACHVGGGTWTCTAAT-3′) [25]. Fungal internal transcribed spacers (ITS) region 1 was amplified using modified ITS1F (5′-CTTGGTCATTTAGAGGAAGTAA-3′) and ITS2 (5′-GCTGCGTTCTTCATCGATGC-3′) [26, 27]. Amplicons for 16S rRNA gene (pair-ended: 150 × 150 bp) and ITS1 region (pair-ended: 250 bp × 250 bp) were sequenced using Illumina MiSeq 500-cycle kit at the Next Generation Sequencing Core (Argonne National Laboratory, Argonne, IL, USA) with the Illumina Miseq sequencing system. The 16S rRNA gene and ITS1 region amplicons were sequenced in separate MiSeq runs.

For bacterial community profiling, the bioinformatics pipeline and subsequent analyses were performed using the R programming language version 4.10. Forward and reverse primers were removed from the MiSeq reads by using the cutadapt plugin v2.10 [28] (DADA2 package). The DADA2 pipeline was used for data processing and taxonomy assignment [29]. Forward and reverse reads were trimmed to 240 base pairs and 200 base pairs, respectively, and at the location of the first occurrence of a base call or containing greater than or equal to 18 estimated errors, and merged with a minimum overlap of 12 base pairs. Chimeric sequences were discarded and merged reads were dereplicated. Taxonomy was assigned to amplicon sequence variants (ASVs) using the SILVA v138 database [30].

Fungal pair-ended amplicon sequences were assembled with QIIME2 2019.4 and reads were generated by demultiplexing. Cutadapt was used for quality filter and removal of adapters and primers. Sequences were further processed with DADA2 and denoise-single-end was performed. The reverse sequences presented low quality; therefore, we proceeded with forward reads. Final ITS sequences ranged from 251 to 474 bases (average 308 bases). Assembled reads were further confirmed to be fungal ITS sequences using UNITE 8.0 [31]. Amplicon sequence variants (ASVs) were assigned at 97% similarity and converted to BIOM file. The cutoff of more than or equal to 10 reads was considered for ASVs included in this study.

### Metagenome sequencing of the wheat rhizosphere

The same DNA sample extracted for amplicon sequencing was used for metagenome sequencing. We merged two biological replicates for metagenome sequencing, resulting in three replicates for each treatment with a final volume of 20 µl (≥ 20 ng µl^−1^) for each sample. Metagenome sequencing library preparation was performed by Novogene Corporation Inc. (California, USA). Sequencing libraries were then prepared using the Nextera kit, according to Illumina’s instructions, on DNA fragments in 350 bp. Barcode and equal molar amount pooled libraries were processed with 150 base pair paired-end sequencing over two rounds, an Illumina MiSeq to check the quality of the DNA and the needed sequencing depth followed by HiSeq 2500. Contamination of reads originating from the host plant was removed by mapping with Bowtie 2.2.5 in sensitive mode against the draft genome of *Triticum aestivum*; paired and unpaired data were stored separately. Reads of all samples were pooled together for an assembly with SPAdes 3.5 using kmers with length 33, 55, 77, 99, and 127 and the ‘careful’ flag enabled. For the resulting contigs, genes were predicted with Prodigal 2.61 in metagenomics mode and stored in General Transfer Format using Cufflinks 2.1.1. All predicted genes were annotated using antiSMASH. We used hmmsearch with all 308 HMMs against all predicted genes in our metagenome. Genes were assigned to taxonomy by running Diamond 0.7.9 and CAT against the non-redundant Blast NCBI database from 20150311. The lowest common ancestor classification was determined using MEGAN 5.10 by taking the top 50% hits and filtering for a minimum score of 50 and maximum expect value of 0.01 and converting the gene identifiers to taxonomy IDs using the mapping provided by MEGAN.

### Bioinformatics and statistical analysis

The ASV tables (16S rRNA gene and ITS region) were rarefied at the lowest sequencing depth obtained from a sample using function rarefy_even_depth from the Phyloseq package (v.1.10) [32]. For the Beta-diversity calculations, the entire filtered ASV table was used and normalized using the function cumNorm from the R package metagenomeSeq. We used a cumulative-sum scaling (CSS) method, which calculates the scaling normalization factors equal to the sum of counts up to a particular quantile to normalize the reads counts, in order to avoid biases generated with current sequencing technologies due to uneven sequencing depth. A Bray–Curtis dissimilarity matrix was calculated and used to build Principal Coordinate Analyses and Constrained Principal Coordinate Analysis constrained by status group, that is, cycle 1, 2, 3, 4, 5, using the function capscale retrieved from the Vegan package (v.2.3-2) [33] and implemented in the Phyloseq package (v.1.10), both in R. The nonparametric adonis test was used to assess the percentage of variation explained by the status grouping along with its statistically significant. Permutational multivariate analyses of variance were performed to evaluate the significance of the constrained principal coordinate analysis, both retrieved from Phyloseq and Vegan packages.

To compare the differences in taxonomic composition (bacterial and fungal taxa) or function composition and to assess whether taxa were differentially abundant, we applied function calculateEffectiveSample from the metagenomeSeq R package to the filtered ASV table or BGCs table and features less than the average number of effective samples in all features were removed. We used normalized tables applying the cumulative-sum scaling normalization as described above. Then, a Zero-inflated Gaussian Distribution Mixture Model was applied to moderated t-tests between accessions using the makeContrasts and eBayes commands retrieved from the R package Limma (v.3.22.7) [34]. Differential abundance analysis of bacterial and fungal communities was performed to identify those dynamic taxa associated with the onset of disease suppression or resistant state.

## Results

### Selection of resistant and susceptible wheat genotypes

We tested 13 wheat genotypes for susceptibility to the root pathogen *B. sorokiniana* and the disease severity index varied from 8.9 to 48.4% (Additional file 1: Figure S3). Based on disease incidence, availability of seeds, and germination homogeneity, we selected contrasting wheat genotypes for a detailed study on disease and microbiome dynamics. Guamirim and Karakilcik were selected as the *Bipolaris*-susceptible genotypes, then Frontana and IAC 5 as the *Bipolaris*-resistant wheat genotypes.

### Disease incidence with repeated exposure of wheat to the fungal root pathogen

**Fig. 1:**
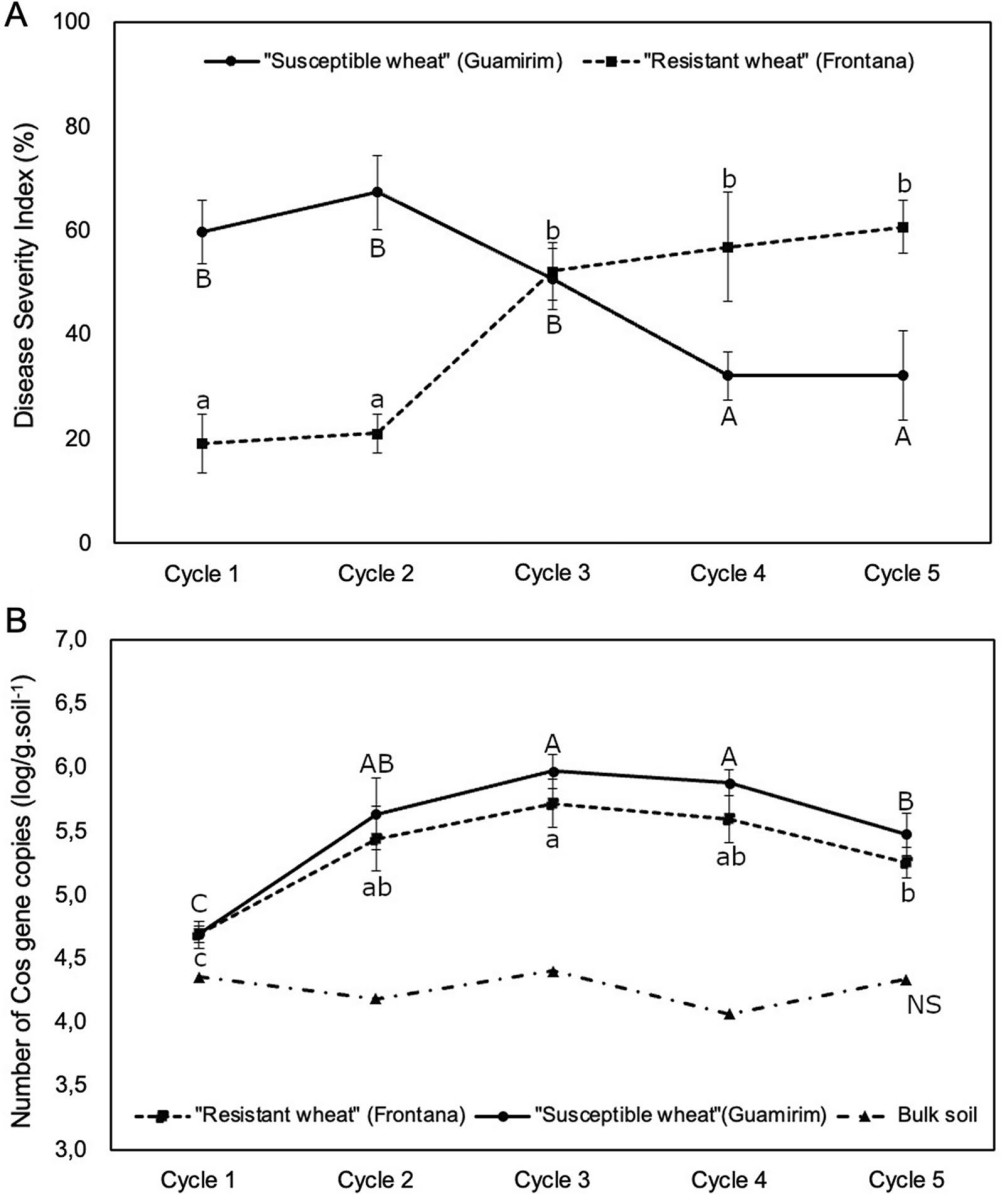
**A** Disease severity index in susceptible (Guamirim) and resistant (Frontana) wheat inoculated with the pathogen *Bipolaris sorokiniana* over 5 successive cultivation cycles of 4 weeks each. **B** qPCR analysis showing the number of Cos gene copies (log/g∙soil^−1^) of the pathogen *B. sorokiniana* on roots of susceptible (Guamirim) and resistant (Frontana) wheat over 5 successive growth cycles. Different letters indicate statistically significant differences across cycles within each treatment (*P* < 0.05, post-hoc Tukey HSD)

Among the four wheat genotypes, Guamirim and Frontana showed the most contrasting patterns in terms of disease onset and development over five successive cultivation cycles. Following exposure to the pathogen, the susceptible genotype Guamirim showed high disease incidence in the first (59.8%) and second (67.4%) growth cycles, followed by a slight reduction in the third (50.8%), and a more substantial drop in disease incidence in the fourth (32.2%) and fifth (32.2%) cycles (Fig. 1A). In contrast, repeated pathogen exposure of the resistant wheat Frontana showed low disease incidence in the first two growth cycles (19.2% and 21.2%, respectively) and, surprisingly, a significant increase in disease incidence in the third (52.3%), fourth (56.9%), and fifth (60.7%) cycles (Fig. 1A). A similar pattern was observed for the other two wheat genotypes tested, that is, disease severity decreased in the susceptible wheat Karakilcik and increased in the resistant wheat IAC 5 over five successive growth cycles (Additional file 1: Figure S4). Although susceptible and resistant wheat showed contrasting patterns, similar densities of the fungal pathogen were detected by qPCR in both conditions (Fig. 1B; Additional file 1: Figure S5). Successive cultivation of all four plant genotypes without pathogen inoculation (control treatments) showed a minor increase in disease severity over time, which is most likely due to infections by indigenous *B. sorokiniana* in the field soil used for the experiment (Additional file 1: Figure S6). To investigate the impact of repeated pathogen exposure on rhizosphere microbiome assembly and functions, we selected the two most responsive and contrasting wheat genotypes, Guamirim (susceptible) and Frontana (resistant), for 16S rRNA gene and ITS region amplicon sequencing and metagenomic profiling.

### Rhizosphere microbiome assembly in the susceptible and resistant wheat

**Fig. 2:**
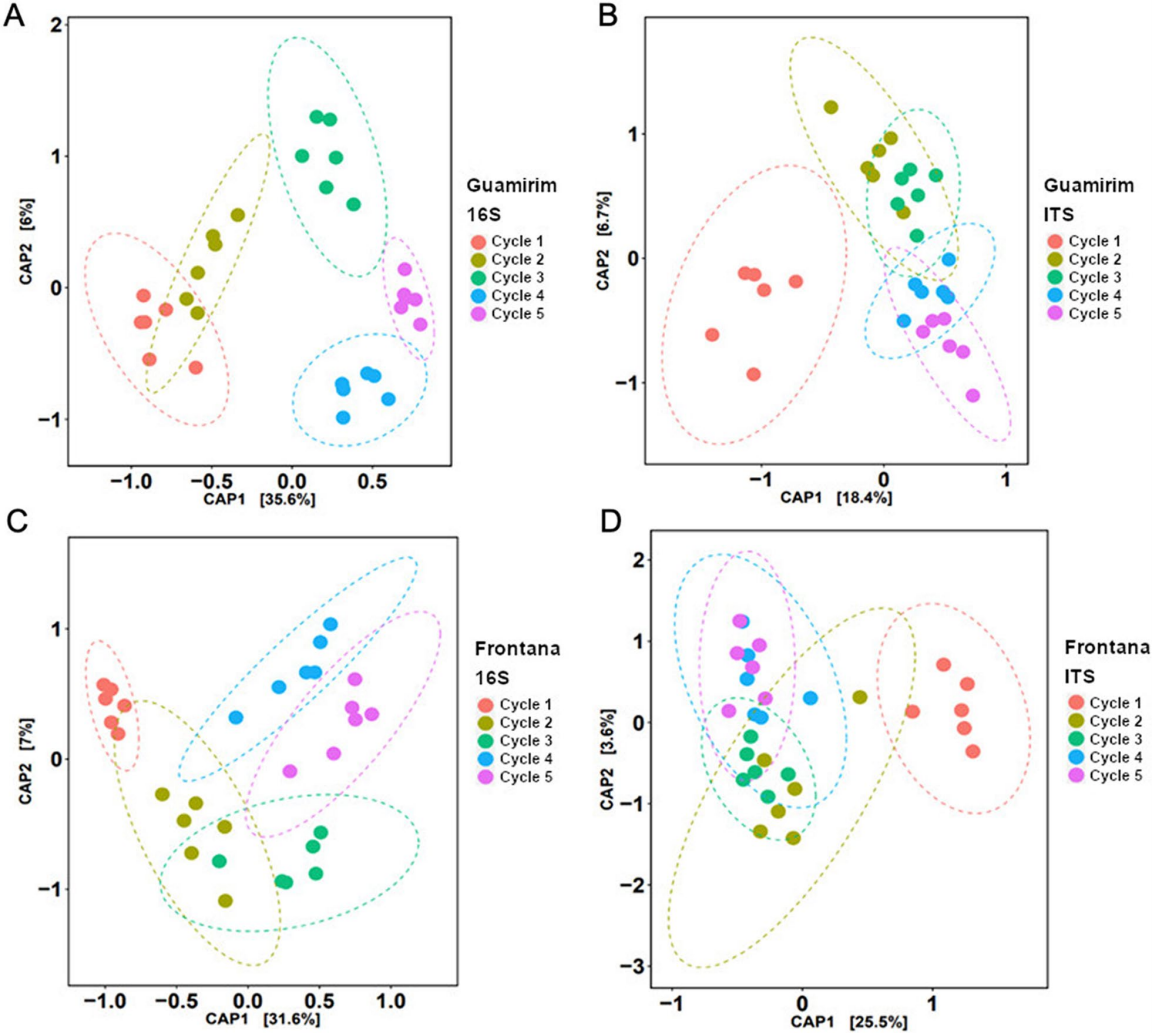
Rhizosphere community structure of susceptible (Guamirim) and resistant (Frontana) wheat cultivars repeatedly exposed to the fungal root pathogen over 5 successive cultivation cycles. Constrained Analysis of Principal Coordinates (CAP) of 16S rRNA (bacteria) or ITS (fungi) amplicon diversity. **A** Bacterial community in the susceptible (Guamirim) wheat rhizosphere; **B** Fungal community in the susceptible (Guamirim) wheat rhizosphere; **C** Bacterial community in the resistant (Frontana) wheat rhizosphere; and **D** Fungal community in the resistant (Frontana) wheat rhizosphere. Statistical differences between growth cycles were calculated with PERMANOVA test (Additional file 1: Table S3, S4, S5 and S6)

Amplicon sequencing analysis showed a pronounced rhizosphere effect on bacterial and fungal community assembly, with the bulk soil samples clustering separately from rhizosphere samples for both wheat genotypes (Additional file 1: Figure S7). Disease-susceptible and disease-resistant wheat exhibited distinct structures in bacterial and fungal communities across all growth cycles (Additional file 1: Figure S7). The susceptible wheat Guamirim presented higher rhizobacterial richness and diversity in growth cycles 1, 2, and 4 (Additional file 1: Figure S8). The overall composition of the bacterial community in each growth cycle is shown in Additional file 1: Figure S9A; it is interesting to note that while the relative abundance of the phylum *Bacteroidota* increased over cycles, the relative abundance of *Actinobacteria* decreased. The beta-diversity of the rhizosphere bacterial communities correlated with the cultivation cycle, as the clustering pattern diverged each cycle (Fig. 2A; Additional file 1: Table S3). The rhizosphere fungal community of the susceptible wheat presented higher diversity and richness in cycle 3 (Additional file 1: Figure S10). The overall composition of the fungal community in each growth cycle is shown in Additional file 1: Figure S9B. The beta-diversity analysis on the rhizosphere fungal communities showed that cultivation cycle 1 clustered separately from the other growth cycles. A homogenous cluster was observed for cycles 2 and 3 and cycles 4 and 5 (Fig. 2B).

The resistant wheat genotype Frontana showed high bacterial diversity in cycles 1, 2, and 4 and higher richness in cycle 1 (Additional file 1: Figure S11). The overall composition of the bacterial community in each growth cycle is shown in Additional file 1: Figure S12A. The beta diversity of the rhizosphere bacterial communities of the resistant plant genotype revealed that cycle 1 and cycle 2 clustered separately from the others cycles (Fig. 2C; Additional file 1: Table S5). For the fungal community, the resistant genotype did not show significant differences (*P* > 0.05) between the cycles for the Shannon diversity index, however presented high richness in the fungal community in cycle 3 (Additional file 1: Figure S13). The overall composition of the fungal community in each growth cycle is shown in Additional file 1: Figure S12B. Furthermore, the structure of the fungal community after cycle 1, did not show any specific clustering pattern over successive cycles (Fig. 2D; Additional file 1: Table S6).

### Identification of bacterial and fungal taxa associated with disease suppression

**Fig. 3:**
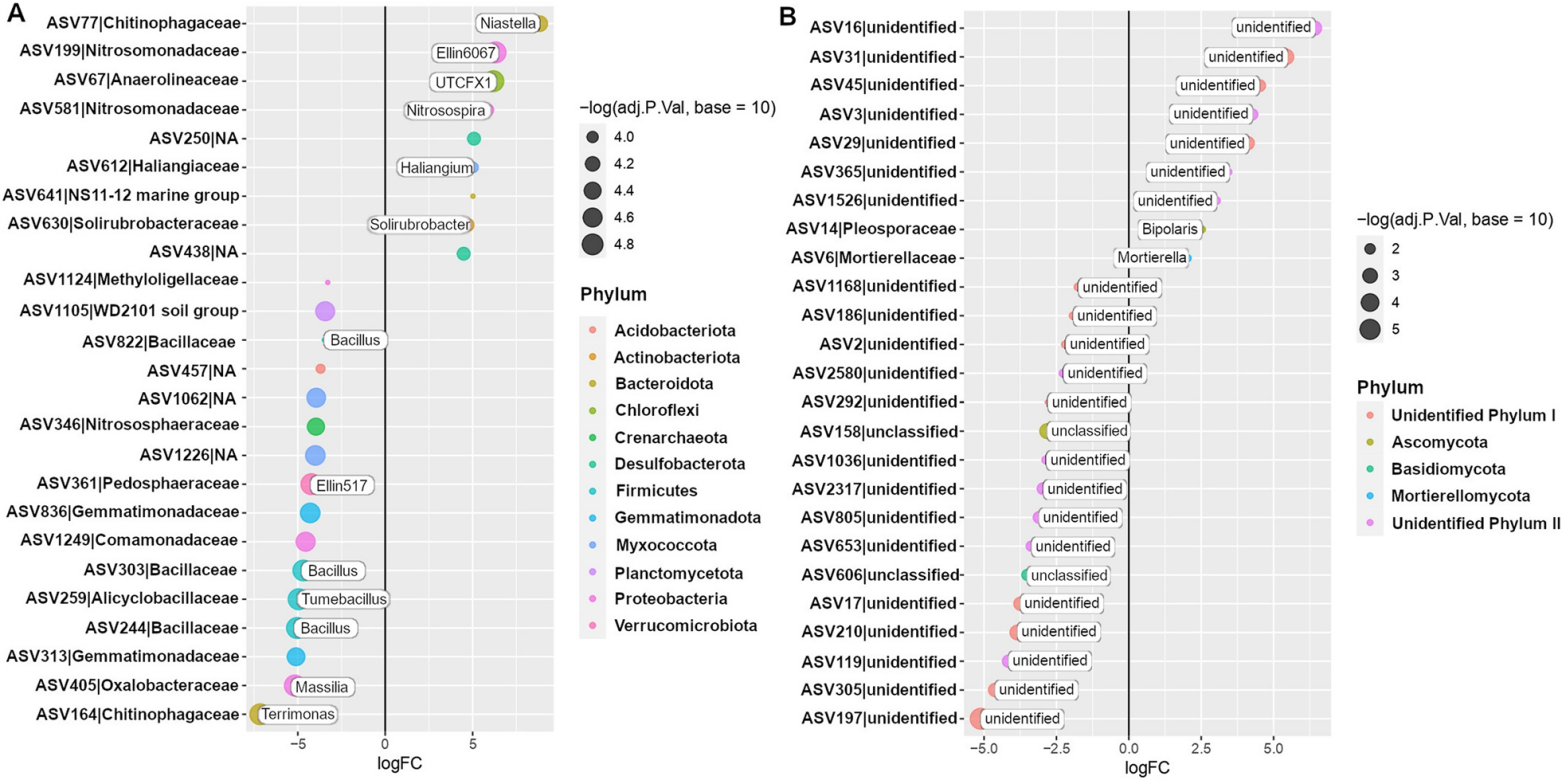
Differentially abundant bacterial (**A**) and fungal (**B**) ASVs identified in the rhizosphere of the *Bipolaris*-susceptible wheat Guamirim. Values on the x-axis show the estimated log2-fold difference in ASVs abundance between cycle 1 and 4, where positive values indicate higher abundance in cycle 4 (low level of disease) and negative values indicate higher abundance in cycle 1 (high level of disease) (FDR adjusted *P* values of < 0.05). Dots indicate ASVs and the size of each dot is scaled by its mean abundance among all samples (base mean > 50). The dot color represents the phylum to which that ASV belongs

**Fig. 4:**
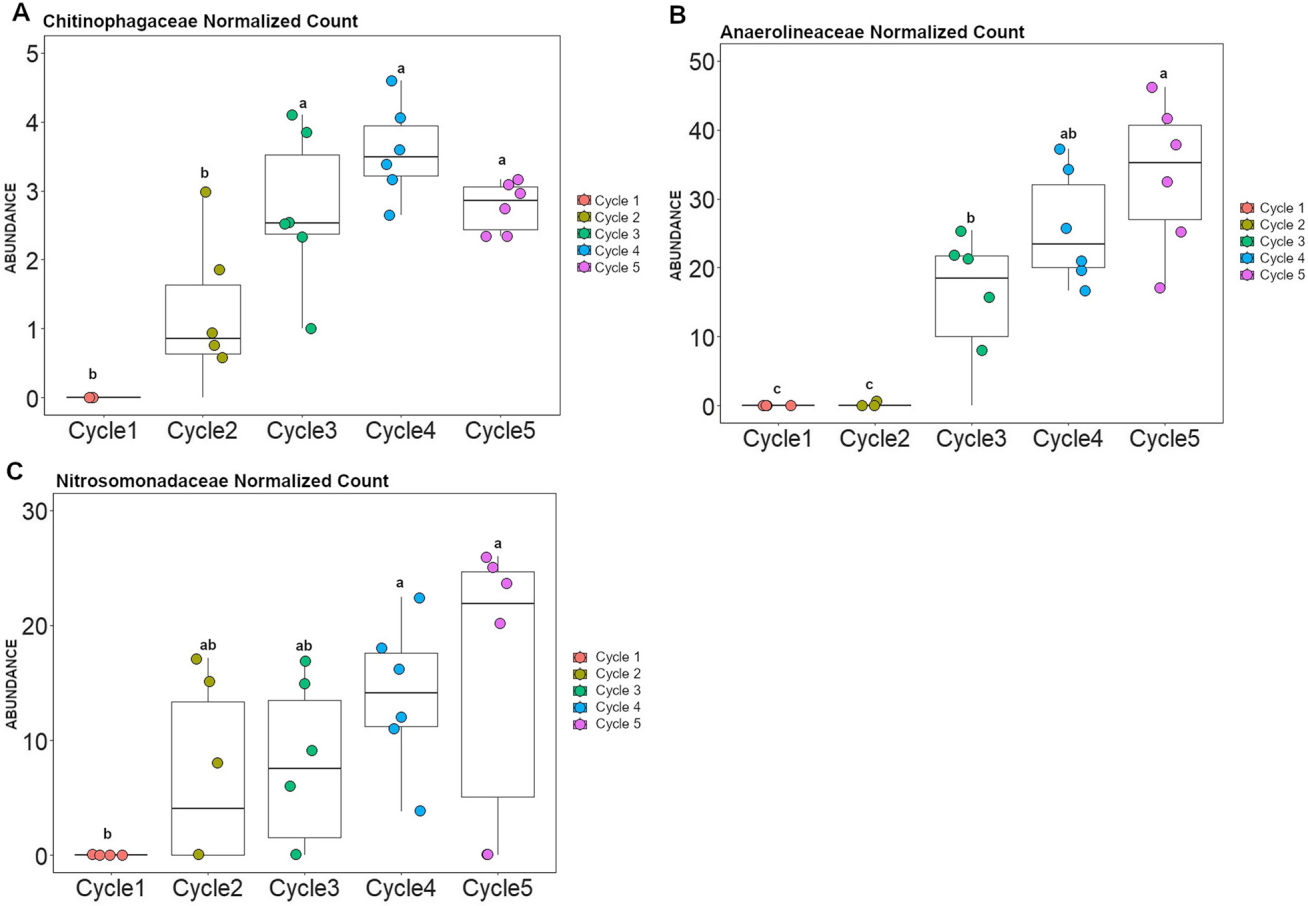
Abundance of bacterial families that increased with disease suppression in the susceptible wheat Guamirim. Dynamics of the bacterial families *Chitinophagaceae* (**A**), *Anaerolineaceae* (**B**) and *Nitrosomonadaceae* (**C**) over five wheat cultivation cycles. Error bars represent the standard deviation of six independent replicates. Different letters indicate significant differences among treatments based on ANOVA post-hoc Tukey HSD (*P* < 0.05)

To identify the bacterial and fungal taxa that are potentially associated with the onset of disease suppressiveness, we selected two growth cycles with contrasting disease severity levels and performed a differential abundance analysis. More specifically, in the susceptible wheat genotype we selected bacterial and fungal taxa that increased in abundance in cycle 4 (low level of disease) in comparison with cycle 1 (high level of disease). The 16S rRNA profiling showed that the phyla *Proteobacteria*, *Bacteroidota* and *Chloroflexi* dominated the enriched bacterial community when the disease was suppressed (Additional file 1: Figure S14). Differential abundance analysis revealed that the top three significantly enriched amplicon sequence variants (ASVs) belonged to *Chitinophagaceae*, *Nitrosomonadaceae*, and *Anaerolineaceae* (Fig. 3A). Then, we selected the top three dynamic ASVs enriched during the onset of disease suppressiveness (cycle 4), i.e., ASV77 (*Chitinophagaceae*), ASV199 (*Nitrosomonadaceae*) and ASV67 (*Anaerolineaceae*) (Fig. 3A), to determine their occurrence over all five cultivation cycles (Additional file 1: Figure S15). In order to determine if the pattern observed for specific ASVs can be extended to their respective bacterial families, we selected all ASVs associated with these three bacterial families and conducted an analysis to validate the dynamics at family level. Interestingly, the abundance of each of these three bacterial families significantly increased with disease suppression over the five cultivation cycles (Fig. 4). For the fungi, all ASVs enriched in cycle 4 were unclassified (Fig. 3B) and the most responsive taxa were represented by ASV16, ASV31, and ASV45 (Fig. 3B and Additional file 1: Figure S16).

**Fig. 5:**
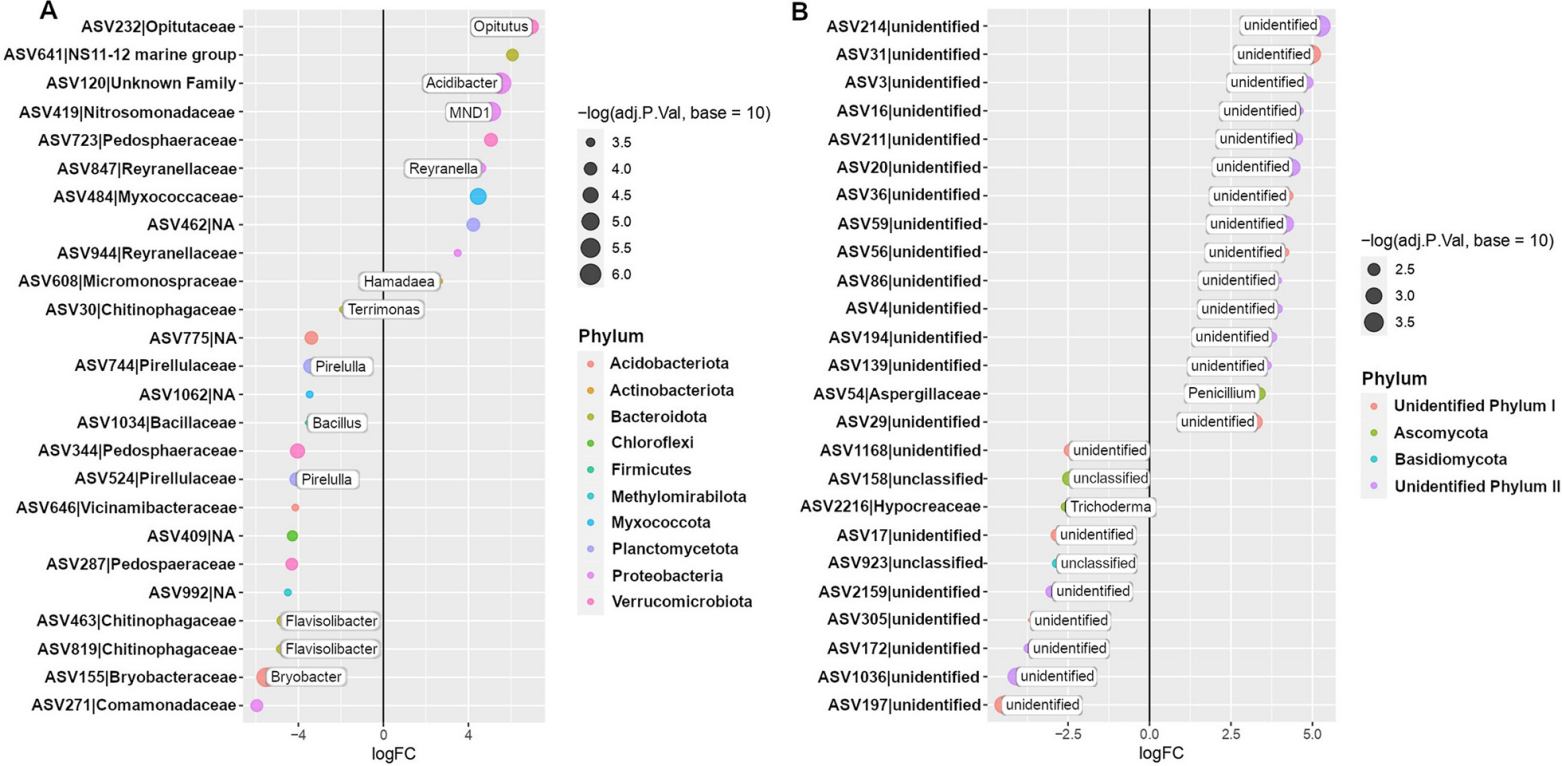
Differentially abundant bacterial (**A**) and fungal (**B**) ASVs identified in the rhizosphere of the *Bipolaris*-resistant wheat cultivar Frontana. Values on the x-axis show the estimated log2-fold difference in ASVs abundance between cycle 1 and 4, where positive values indicate higher abundance in cycle 4 (high level of disease) and negative values indicate higher abundance in cycle 1 (low level of disease) (FDR adjusted *P* values of < 0.05). Dots indicate ASVs and the size of each dot is scaled by its mean abundance among all samples (base mean > 50). The dot color represents the phylum to which that ASV belongs

**Fig. 6:**
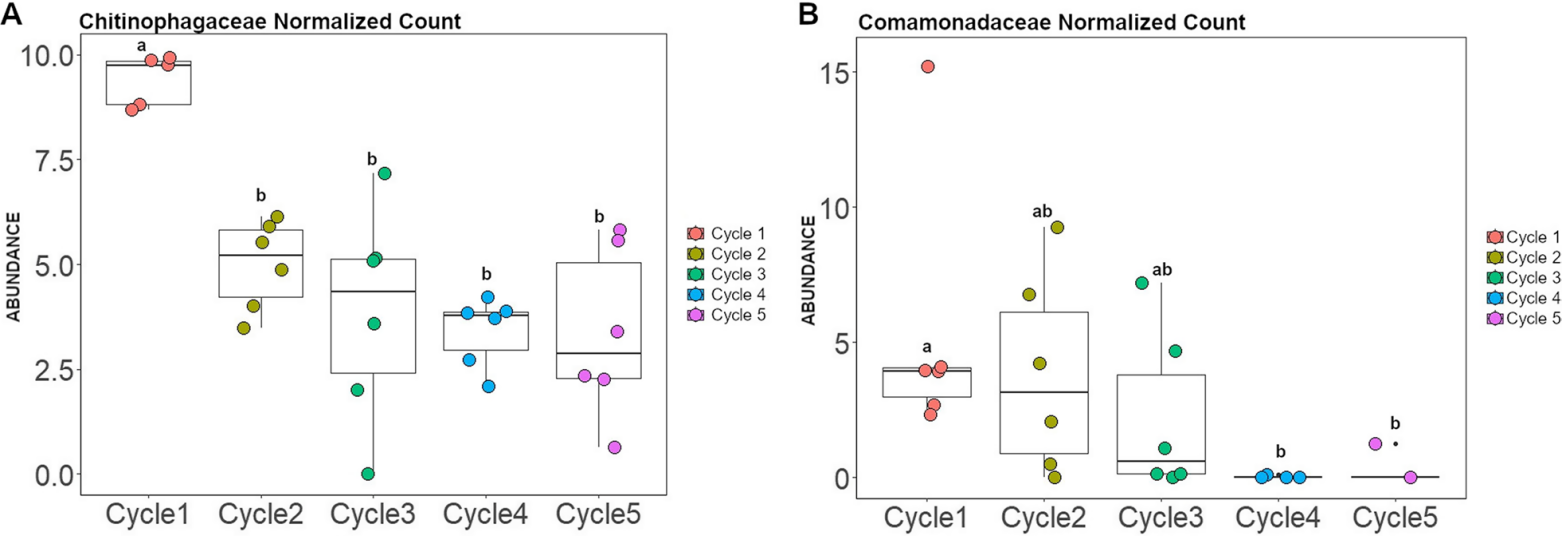
Abundance of bacterial families that decreased with disease progression over plant growth cycles in the resistant wheat Frontana. Dynamics of the bacterial families *Chitinophagaceae* (**A**) and *Comamonadaceae* (**B**) over five wheat cultivation cycles. Error bars represent the standard deviation of six independent replicates. Different letters indicate significant differences among treatments based on ANOVA post-hoc Tukey HSD (*P* < 0.05)

To identify members of the rhizosphere microbiome potentially associated with disease suppression in the resistant wheat genotype, we followed a similar reverse approach as described above comparing the microbiome composition of the first cycle (low level of disease) with cycle 4 (high level of disease). The 16S rRNA profiling showed that *Proteobacteria*, *Bacteroidota* and *Acidobacteriota* dominated the enriched bacterial community in low levels of disease (Additional file 1: Figure S17). Differential abundance analysis revealed that the top three enriched ASVs belonged to *Comamonadaceae*, *Bryobacteraceae* and *Chitinophagaceae* (Fig. 5A). Then, we selected the top three dynamic ASVs more abundant in cycle 1 (low disease severity), i.e., ASV271 (*Comamonadaceae*), ASV155 (*Bryobacteraceae*) and ASV819 (*Chitinophagaceae*) (Fig. 5A), to assess their occurrence over all five cultivation cycles (Additional file 1: Figure S18). Using the same approach applied for the susceptible wheat, we selected all ASVs associated with these bacterial families to validate the pattern observed for specific ASVs at the family level. The abundance of the *Chitinophagaceae* and *Comamonadaceae* families were higher in the plant growth cycles with low disease severity and significantly decreased with the progress of the disease over cycles (Fig. 6). The ITS profiling, showed that all ASVs enriched in cycle 1 were unclassified (Fig. 5B) and the most responsive fungal taxa were ASV197, ASV1036 and ASV172 (Additional file 1: Figure S19).

### Microbiome functionalities in the susceptible wheat genotype

**Fig. 7:**
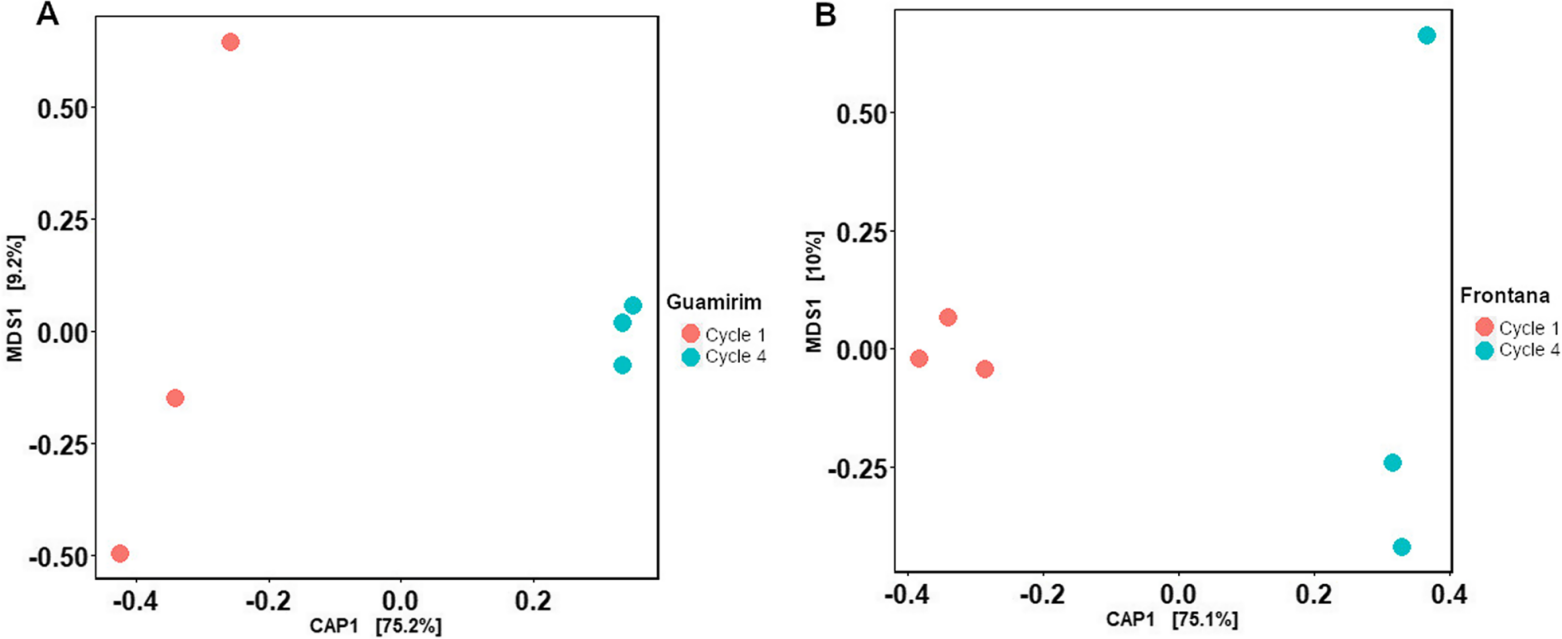
Functional gene profile obtained from metagenome data in the rhizosphere microbiome of the susceptible wheat Guamirim (**A**) and resistant wheat Frontana (**B**). Constrained Analysis of Principal Coordinates (CAP) of functional gene diversity comparing cycle 1 and cycle 4. Statistical significance of the constrained analysis was assessed by ANOVA, *P* < 0.01 for all presented data

**Fig. 8:**
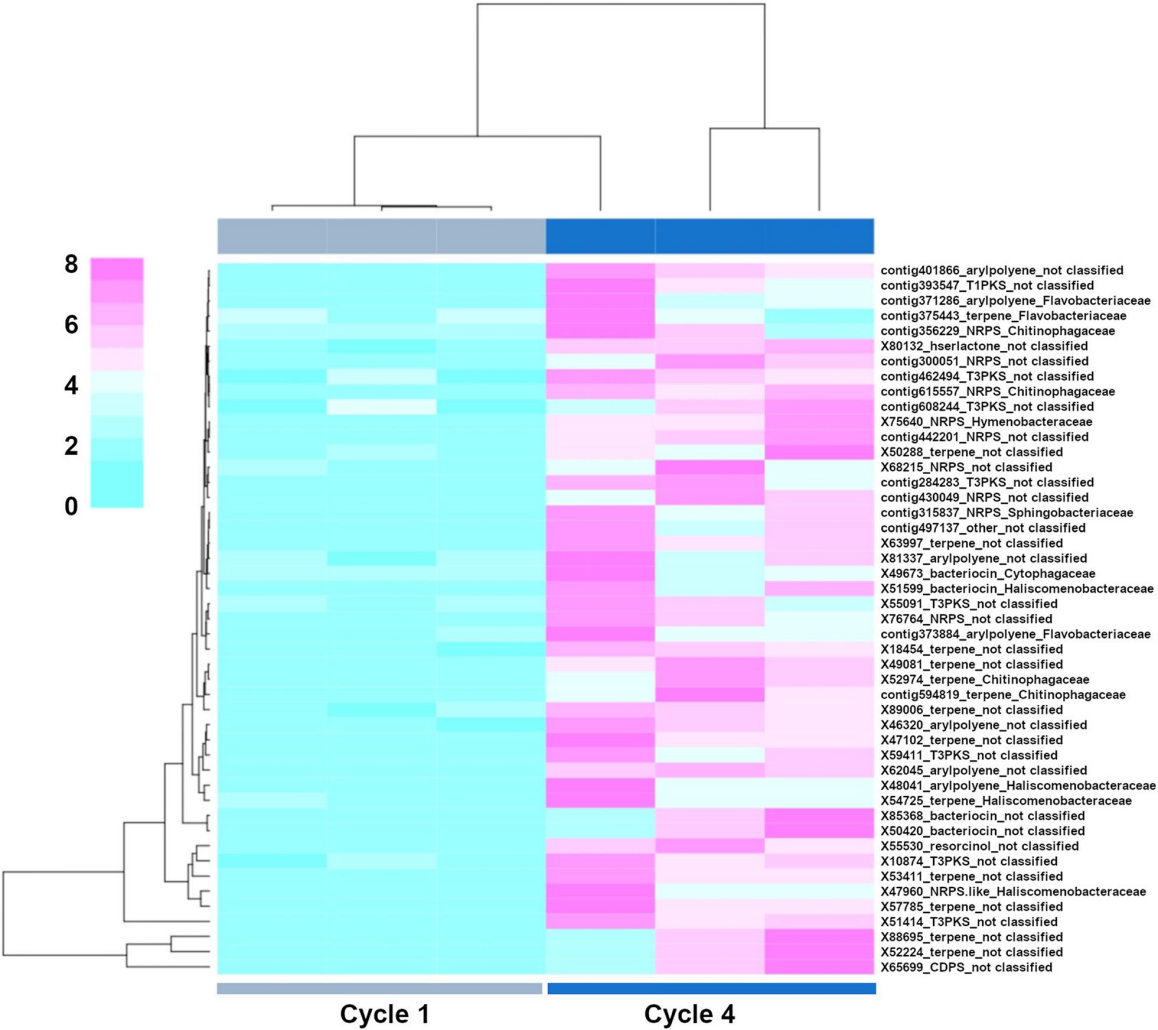
Clustered heat map of relative abundance (normalized counts) of the 47 gene clusters significantly overrepresented in the susceptible wheat Guamirim, cultivation cycles 1 and 4 with three replicates. The cluster number and the corresponding taxonomic assignment (*Bacteroidota*) are shown on the right side of the panel

Based on the amplicon sequencing data analysis, we selected cycles 1 and 4 for metagenome sequencing and functional analysis. The functional annotation of the metagenome data for the susceptible wheat Guamirim revealed different functional profiles between cycle 1 (high level of disease) and cycle 4 (low level of disease) (Fig. 7A). AntiSMASH analysis revealed a total of 2,571 BGCs of which 604 BGCs were significantly overrepresented in the rhizosphere of the susceptible wheat genotype in cycle 4 (low disease) when compared to cycle 1 (high disease) (Additional file 1: Figure S20). These BGCs were grouped in 32 classes with the most overrepresented BGCs associated with the biosynthesis of terpenes, non-ribosomal peptides, polyketides, aryl polyenes, and post-translationally modified peptides (RiPPS) (Additional file 1: Figure S21). Notably, terpene BGCs were also overrepresented in those taxa enriched in the rhizobacterial community in cycle 4, with 17 terpenes for *Proteobacteria* species, 14 terpenes for *Bacteroidota*, and 13 terpenes for *Chloroflexi* (Additional file 1: Figure S22). Considering that the abundance of *Bacteroidota* is positively correlated with disease suppression, and that this phylum harbors *Chitinophagaceae*, which was the bacterial family taxa associated with low disease severity in both wheat genotypes (Figs. 4 and 5), we targeted the *Bacteroidota* for a more detailed analysis. Altogether, 47 BGCs belonging to *Bacteroidota*, including terpenes, NRPS, aryl polyene, and polyketides significantly increased in abundance from cycle 1 to cycle 4 in the susceptible wheat genotype (Fig. 8). For the *Bacteroidota* species, 9 NRPS, 14 terpenes, and 7 aryl polyenes BGCs were overrepresented in cycle 4, belonging to *Chitinophagaceae*, *Flavobacteriaceae*, and *Sphingobacteriaceae* (Fig. 8).

### Microbiome functionalities in the resistant wheat genotype

**Fig. 9:**
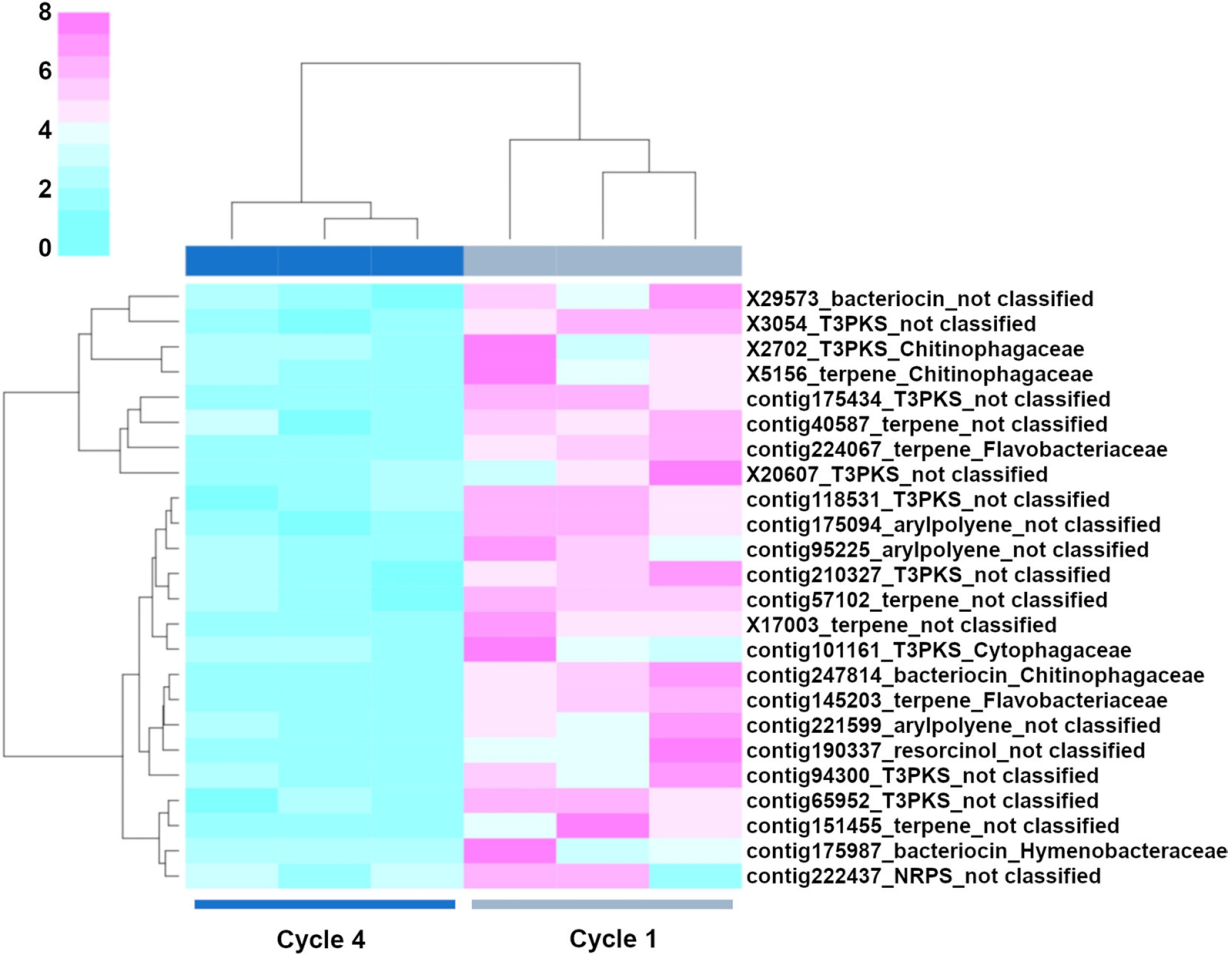
Clustered heat map of relative abundance (normalized counts) of the 26 gene clusters significantly overrepresented in the resistant wheat Frontana, cultivation cycles 1 and 4 with three replicates. The cluster number and the corresponding taxonomic assignment (*Bacteroidota*) are shown on the right side of the panel

The functional annotation of the metagenome data for the resistant wheat genotype Frontana revealed differences between cultivation cycle 1 (low level of disease) and cycle 4 (high level of disease) (Fig. 7B). To identify those BGCs potentially associated with plant protection we targeted BGCs abundant in cycle 1 (low level of disease) and those depleted in cycle 4 (high level of disease) (C1 > C4). A total of 526 BGCs was found to be significantly overrepresented in the resistant genotype rhizosphere in cycle 1 when compared to cycle 4 (Additional file 1: Figure S23). 31 classes of BGCs were enriched in cycle 1, with the most overrepresented BGCs associated with the biosynthesis of terpenes, non-ribosomal peptides, polyketides, aryl polyenes, and post-translationally modified peptides (RiPPS) (Additional file 1: Figure S24). Terpene BGCs were overrepresented in those taxa enriched in the rhizobacterial community, with 31 terpenes for the *Proteobacteria* species, 7 terpenes for the *Bacteroidota*, and 4 terpenes for the *Acidobacteria* (Additional file 1: Figure S25). Similar to the susceptible wheat genotype, we also targeted the *Bacteroidota* phylum. Altogether, 26 BGCs belonging to *Bacteroidota*, with several terpenes, NRPS, aryl polyene and polyketides, were significantly depleted in cycle 4 when compared with cycle 1 (Fig. 9). For the *Bacteroidota* species, 1 NRPS, 7 terpene, 7 polyketides (type 3) and 7 aryl polyene gene clusters were overrepresented in cycle 1, belonging to *Chitinophagaceae*, *Flavobacteriaceae* and *Cytophagaceae*, respectively (Fig. 9).

## Discussion

In contrast to above-ground plant pathogens, plant genetic resistance to root diseases is much less common. Studying the wheat root disease "take-all", Cook et al. [35] suggested that plants have developed a different strategy to counteract soilborne pathogens by recruiting and activating specific rhizosphere microbiome members for protection. The process of microbial communities’ assembly in the rhizosphere hinges on the interplay between soil type and plant genotype [36]. Remarkably, prior investigations have underscored the pivotal role of diverse plant genotypes in shaping the assembly of bacteria [16, 18], fungi and protists [18], as well as viral communities [37]. In previous studies on soils suppressive to *Rhizoctonia solani*, we confirmed the microbiological nature of plant protection [3, 10, 13]. We also demonstrated that the host plant relies on the composition and activity of the rhizosphere [3] and endosphere [10] microbiomes to fend off soil-borne pathogens. In these previous studies, we assessed the microbiome in a single cultivation cycle with soil that was already in a disease suppressive state. Here, we used pot experiments to induce disease suppressiveness in a conducive soil under lab conditions over five growth cycles of wheat in presence of the pathogen *B. sorokiniana*. For the susceptible wheat genotype, we showed a significant reduction in disease incidence following successive growth in presence of the pathogen. However, infection of the resistant wheat resulted in a reduction in disease suppression after five growth cycles, the opposite pattern observed for the susceptible wheat. Quantitative PCR analysis revealed similar dynamics of the pathogen in the rhizosphere of both systems yet contrasting conditions, indicating that the susceptible wheat cultivar under suppressive conditions is able to thrive in the presence of high densities of the virulent pathogen. Unexpectedly, increasing levels of disease were observed in later cultivation cycles of the *Bipolaris*-resistant wheat. This unexpected pattern suggests that the pathogen pressure over cycles is able to disrupt the microbiome structure making the rhizosphere more conducive to the pathogen. Considering that the virulent pathogen was inoculated prior to each growth cycle, changes in virulence of the inoculum may have occurred during the course of the successive cultivation allowing the pathogen to overcome the resistance in wheat. Re-isolation, genomic and phenotypic characterization of the pathogen over the course of the successive cycling will be needed to support this hypothesis. Moreover, elucidating the intrinsic resistance basis of this plant genotype would also be important to draw conclusions on pathogen-microbiome interactions. Despite these confounding factors, the significant correlation between the structure of the rhizobacterial communities and the dynamics of disease severity across cultivation cycles, also suggested that the resistant wheat cultivar is, to some extent, dependent on the rhizosphere microbiome to counteract pathogen infection.

For the susceptible wheat genotype, the alpha diversity of the bacterial community was higher at high levels of disease, whereas an opposite pattern was observed for the resistant wheat with a higher alpha bacterial diversity at low levels of disease. These patterns suggest that the pathogen either directly or indirectly via root infection disrupted the rhizosphere microbiome affecting the diversity with concomitant changes in their interactions with the pathogen. Although disease suppression in disease suppressive soils is frequently associated with changes in rhizosphere microbiome [3, 13], these changes most likely occur in concert with other plant defense mechanisms activated during fungal invasion. For example, enrichment of beneficial microbes in the rhizosphere may boost the capacity of plant defense thru induced systemic resistance (ISR) [12].

To identify members of the microbiome associated with plant protection, we conducted a differential abundance analysis of the microbiomes in cycles where disease incidence was high with those cycles where disease incidence was low. The dynamics of the identified differential taxa was then assessed for all growth cycles. The bacterial families *Chitinophagaceae*, *Nitrosomonadaceae*, and *Anaerolineaceae* showed a negative correlation with disease incidence in the susceptible wheat, i.e., they consistently increased in abundance as disease levels decreased over repeated growth cycles. In the resistant wheat, the bacterial families *Chitinophagaceae* and *Comamonadaceae* were negatively correlated with disease incidence. Integrating both yet contrasting data sets revealed that *Chitinophagaceae* is consistently associated with low levels of disease in both wheat genotypes. This bacterial family was also reported to play a key role in disease suppression against *R. solani* in sugar beet [10], *Fusarium oxysporum* in banana [38], *R. solani* AG8 in wheat [39] and *Ralstonia solanacearum* in tomato [40].

Next to the role of rhizosphere bacteria in disease suppressiveness, a highly diverse plant-associated fungal community is crucial for plant health [41]. Interestingly, the most dynamic fungal ASVs that correlated with disease suppression, in the susceptible (10 ASVs) and resistant (21 ASVs) wheat cultivars, were categorized as “Unclassified”. The yet incomplete taxonomic classification of fungi detected in microbiome studies precludes a more in-depth analysis of their roles in microbiome-associated phenotypes. Nevertheless, in a recent study comparing pepper plants infected or not with *Fusarium*, the authors showed that the fungal community is more sensitive to *Fusarium* wilt than the bacterial community associated to the plant [42]. This finding reinforces the importance of the fungal community in the disease onset.

Metagenome analysis of the rhizosphere microbiome of the susceptible wheat genotype revealed a significant number of BGCs associated with terpenes overrepresented during the onset of disease suppressiveness. Genes for terpene biosynthesis are present in a large number of genomes of plant-associated bacteria and have diverse ecological functions, including stress alleviation, host growth promotion, and defense against pathogens as well as functioning as chemical defense against herbivores and pathogens [43, 44]. Bacterial terpenes are implicated in interkingdom signaling, as these volatile compounds elicit responses from plants [45]. Terpenes were also found to be associated with the suppression of *Phytophthora capsici* in the *Foeniculum vulgare* rhizosphere [46]. Terpenes BGCs identified in our study were affiliated to a number of bacterial taxa responsive to pathogen exposure, as pointed by amplicon sequencing data. These again include *Chitinophagaceae*, reinforcing their potential role in plant protection and is consistent with the results obtained in wheat plants infected with *R. solani* AG8, where *Chitinophaga* and also *Flavobacterium* increased in abundance in the wheat rhizosphere after nine cultivation cycles [39].

The fungal cell wall composed of branched β-glucan-chitin represents a carbohydrate armour for pathogens [47]. Therefore, activity of chitin-degrading enzymes in the root microbiome during fungal invasion, can result in plant protection against soil borne pathogens as exemplified by Carrión et al. [10]. Pathogen-induced activation of *Chitinophagaceae* containing several enzymes associated with fungal cell wall degradation, including chitinases, β-glucanases and endoglucanases contributed to disease suppression of *R. solani* in the sugar beet endophytic root microbiome [10]. Indeed, the genetic potential of bacteria to synthesize different types of natural products influences microbial interactions. Besides, specific functional traits related to pathogen suppression, for instance, protein secretion system and antifungal compounds are more abundant in the bacterial rhizosphere community of disease-resistant varieties of common bean than in susceptible varieties [48].

Although a better understanding of how these compounds are produced and regulated in the rhizosphere microbiome is needed, these BGCs are involved in niche adaptation and represent an intense microbial arms race that controls microbial community composition and activity. These findings in our study suggest that bacterial community structure can be trait-based assembled favoring microbiome members that provide plant protection over planting cycles resulting in disease suppression.

We have demonstrated how compositional and functional changes were induced in the rhizosphere microbiome upon pathogen exposure leading to disease suppressiveness. However, the knowledge on the mechanisms underlying the interactions between the rhizosphere microbiome with susceptible versus resistant plant genotypes is limited. Future experiments will be directed to address if disease suppression is returned when soils are exchanged between both wheat genotypes after repeated exposure. In conclusion, we combined taxonomic and functional profiling to identify key changes in the rhizosphere microbiome during disease suppression. This illustrates how the host plant relies on the rhizosphere microbiome as the first line of defense to fight soil-borne pathogens. Exploring ways to boost protective members of the rhizosphere microbiome represents a promising strategy to enhance the natural defense mechanism that emerges from the interaction between the host plant, pathogen, and the microbiome.

### Supplementary Information

**Additional file 1.** Supplementary material.

## Data Availability

The datasets generated and/or analysed during the current study are available in the EMBL-EBI repository, project number PRJEB62483.
