# Supplementary material for: Repeated exposure of wheat to the fungal root pathogen Bipolaris sorokiniana modulates rhizosphere microbiome assembly and disease suppressiveness
Source: Environ Microbiome. 2023 Dec 5;18:85. doi: 10.1186/s40793-023-00529-2 (PMC10696838; doi:10.1186/s40793-023-00529-2)
Supplement: Supplementary file 1 — Additional file 1. Supplementary material. [file 40793_2023_529_MOESM1_ESM.pdf]

SUPPLEMENTARY MATERIAL

Repeated exposure of wheat to the fungal root pathogen *Bipolaris sorokiniana* modulates rhizosphere microbiome assembly and disease suppressiveness

Lilian S Abreu Soares Costa[1,3]; Mírian Rabelo de Faria[1]; Josiane Barros Chiaramonte[1]; Lucas W Mendes[2]; Edis Sepo[3]; Mattias de Hollander[3]; José Maurício Cunha Fernandes[4]; Víctor J. Carrión[3,5,6,7]; Wagner Bettiol[1]; Tim H Mauchline[8], Jos M Raaijmakers[3,5], Rodrigo Mendes[1*].

[1]*Embrapa Environment, Jaguariúna, Brazil;* [2]*Center for Nuclear Energy in Agriculture, University of são Paulo, Piracicaba, Brazil;* [3]*Department of Microbial Ecology, Netherlands Institute of Ecology (NIOO-KNAW), The Netherlands;* [4]*Embrapa Wheat, Passo Fundo, Brazil;* [5]*Institute of Biology, Leiden University, The Netherlands;* [6]*Departamento de Microbiología, Facultad de Ciencias, Universidad de Málaga, Spain;* [7]*Departamento de Microbiología y Protección de Cultivos, Instituto de Hortofruticultura Subtropical y Mediterránea "La Mayora", Universidad de Málaga-Consejo Superior de Investigaciones Científicas, Málaga, Spain;* [8]*Sustainable Soils and Crops, Rothamsted Research, Harpenden UK.*

**Corresponding author* rodrigo.mendes@embrapa.br

This file contains:

Supplementary Tables 1 to 6

Supplementary Figures 1 to 25

Supplementary Table 1 Chemical analyses of the soil used in the plant bioassay.

B (mg dm^{-3})	Cu (mg dm^{-3})	Fe (mg dm^{-3})	Mn (mg dm^{-3})	Zn (mg dm^{-3})	P Resin (mg dm^{-3})	N total (mg kg^{-1})
0.46	6.6	12	46.2	2.9	49	3500
K Resin (mmolc dm^{-3})	Ca Resin (mmolc dm^{-3})	Mg Resin (mmolc dm^{-3})	OM (g kg^{-1})	OC (g kg^{-1})	pH CaCl2	OM (g dm^{-3})
8	60	43	25	14	5.7	31
H+Al (mmolc dm^{-3})	SB (mmolc dm^{-3})	CTC (mmolc dm^{-3})	V %	Al KCl 1 mol L^{-1} (mmolc dm^{-3})	m %	
18	111	129	86	<0.02	0	

Supplementary Table 2. Information on the wheat genotypes used in the study.

**Genotype**	**GB access code**[1]	**GB classification**[2]	**Country of origin**	**Launch date**
Karakilcik PI 341349[4]	BGT 05139/1	Landrace	Turkey	NIA[3]
Pakintan 81	BGT 15185/2	Landrace	Pakistan	NIA
BH 1146	BGT 01301/2	Modern cultivar	Brazil	1955
Frontana[5]	BGT 03680/6	Modern cultivar	Brazil	1940
IAC 5-Maringá	BGT 04434/1	Modern cultivar	Brazil	1969
BRS Guamirim[4]	BGT 11416/3	Modern cultivar	Brazil	2005
Quartzo	BGT 15078/1	Modern cultivar	Brazil	2008
Sumai 3	-	Modern cultivar	China	1979
Toropi	-	Modern cultivar	Brazil	1965
CEP24 Industrial	-	Modern cultivar	Brazil	1992
BR 18 Terena	-	Modern cultivar	Brazil	1986
IAC 385[5]	-	Modern cultivar	Brazil	2012
BRS 194	-	Modern cultivar	Brazil	2000

[1]Germplasm Bank of Embrapa Wheat access code;

[2]Germplasm Bank of Embrapa Wheat classification;

[3]No Information Available;

[4]Wheat genotype selected as Bipolaris-resistant:

[5]Wheat genotype selected as Bipolaris-susceptible.

Table 3 Permanova and pairwise analyses comparing bacterial community structures across planting cycles in the susceptible wheat Guamirim inoculated with the pathogen *Bipolaris sorokiniana*.

**Genotype**	**Factor**	***F* value**	**r^2**	***p* value**	
**Guamirim**	Cycles	6.30	0.50	0.01	
**Pairwise (*p* value)**	C2	C1	C3	C4	C5
C2	-	0.0033	0.0025	0.0021	0.0017
C1	0.0033	-	0.0021	0.0018	0.0022
C3	0.0025	0.0021	-	0.0018	0.0035
C4	0.0021	0.0018	0.0018	-	0.0015
C5	0.0017	0.0022	0.0035	0.0015	-

Pairwise *p* value, sequential Bonferroni significance.

Table 4 Permanova and pairwise analyses comparing fungal community structures across planting cycles in the susceptible wheat Guamirim inoculated with the pathogen *Bipolaris sorokiniana*.

**Genotype**	**Factor**	***F* value**	**r^2**	***p* value**	
**Guamirim**	Cycles	2.74	0.30	0.01	
**Pairwise (*p* value)**	C2	C3	C4	C5	C1
C2	-	**0.1165**	0.0093	0.002	0.003
C3	**0.1165**	-	**0.2983**	0.0168	0.0028
C4	0.0093	**0.2983**	-	**0.6919**	0.0015
C5	0.002	0.0168	**0.6919**	-	0.0027
C1	0.003	0.0028	0.0015	0.0027	-

Pairwise *p* value, sequential Bonferroni significance.

Table 5 Permanova and pairwise analyses comparing bacterial community structures across planting cycles in the resistant wheat Frontana inoculated with the pathogen *Bipolaris sorokiniana*.

**Genotype**	**Factor**	***F* value**	r^2	***p* value**	
**Frontana**	Cycles	5.25	0.45	0.01	
**Pairwise (*p* value)**	C2	C1	C3	C4	C5
C2	-	0.0023	0.0067	0.002	0.0025
C1	0.0023	-	0.0021	0.0029	0.0023
C3	0.0067	0.0021	-	**0.0114**	**0.023**
C4	0.002	0.0029	**0.0114**	-	**0.0351**
C5	0.0025	0.0023	**0.0235**	**0.0351**	-

Pairwise *p* value, sequential Bonferroni significance.

Table 6 Permanova and pairwise analyses comparing fungal community structures across planting cycles in the resistant wheat Frontana inoculated with the pathogen *Bipolaris sorokiniana*.

**Genotype**	**Factor**	***F* value**	r^2	***p* value**	
**Frontana**	Cycles	3.20	0.33	0.01	
**Pairwise (*p* value)**	C2	C3	C4	C5	C1
C2	-	**0.2926**	**0.3478**	**0.0924**	0.0025
C3	**0.2926**	-	**0.5034**	**0.1594**	0.0022
C4	**0.3478**	**0.5034**	-	**0.8801**	0.0026
C5	**0.0924**	**0.1594**	**0.8801**	-	0.0016
C1	0.0025	0.0022	0.0026	0.0016	-

Pairwise *p* value, sequential Bonferroni significance.

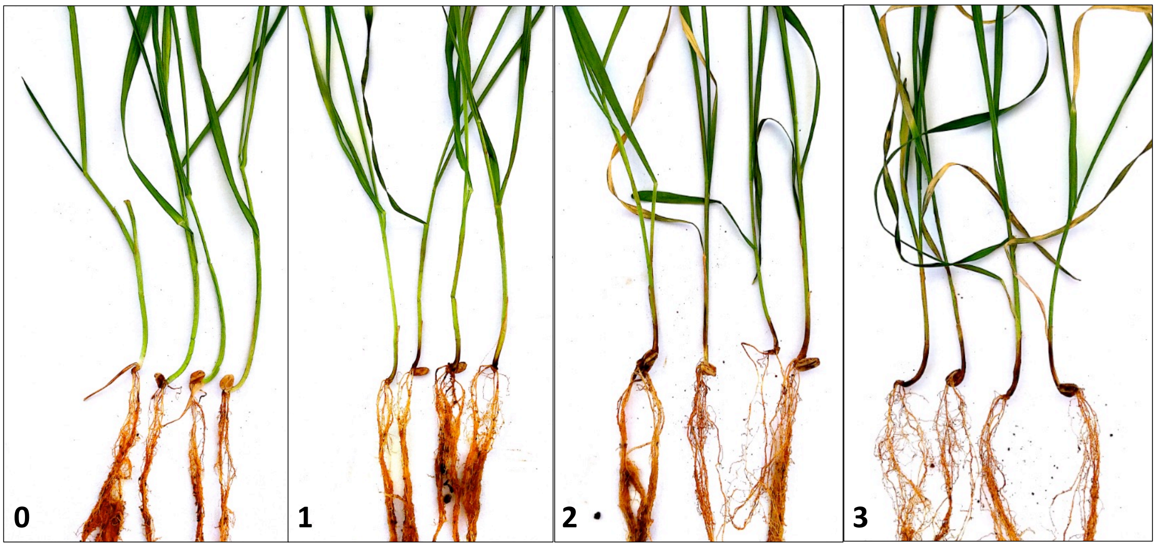

Supplementary Figure 1 Disease severity index ranging from 0 (heathy plant) to 3 (severe symptoms): 0 = no symptoms, 1 = infected plants with slight dark lesion (only on the cotyledon leaf), 2 = infected plants with moderate dark or red lesion on the stem, 3 = severe dark symptoms on the stem and above the first leaf. The number of infected plants were scored 4 weeks after inoculation.

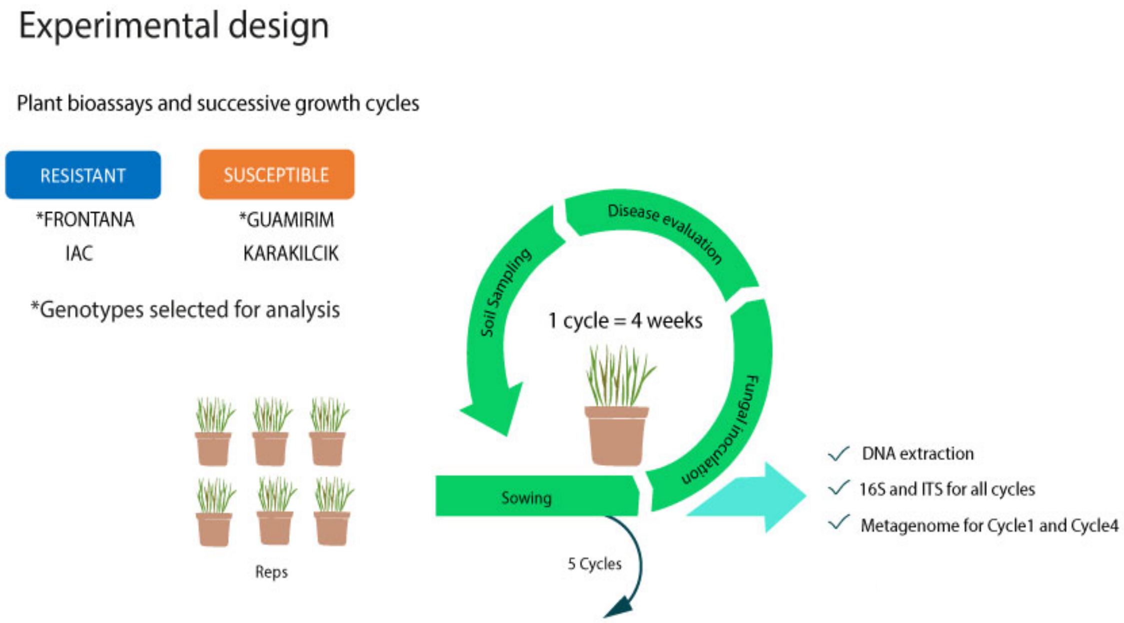

Supplementary Figure 2 Schematic representation of the experimental design and analyses performed for the rhizosphere microbiome assessment in wheat.

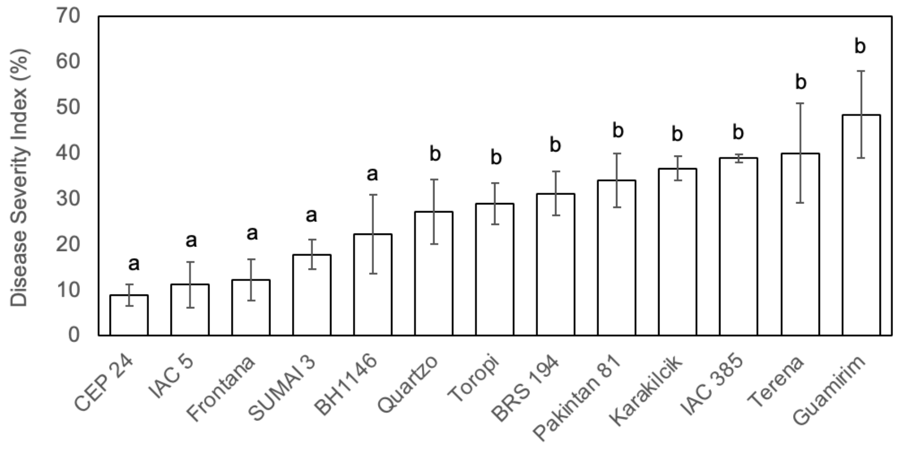

Supplementary Figure 3 Disease severity index on wheat genotypes inoculated with *Bipolaris sorokiniana*. Means with the same letter are not significantly different according to the Scott and knott's test (P < 0.05). Bars represent the standard error of mean.

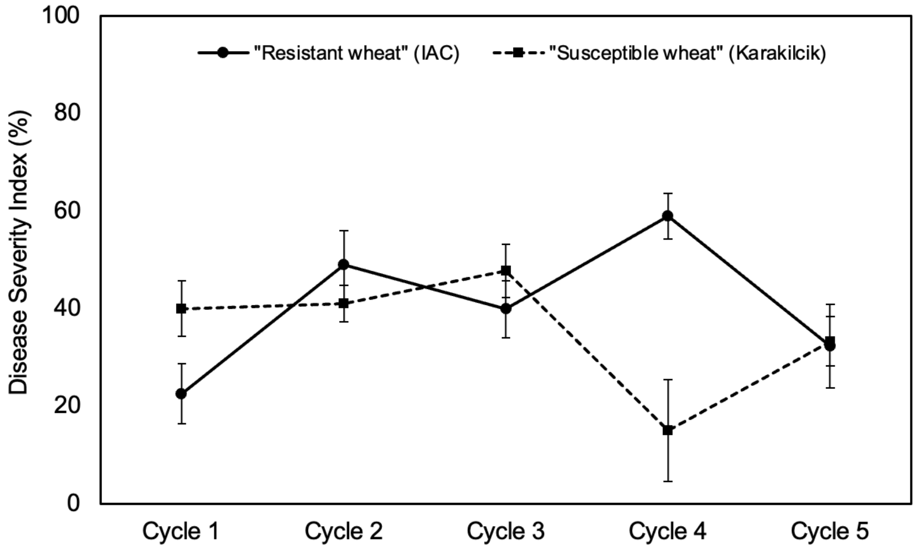

Supplementary Figure 4. Disease dynamics in resistant (IAC 5) or susceptible (Karakilcik) wheat infected with *Bipolaris sorokiniana* over five planting cycles.

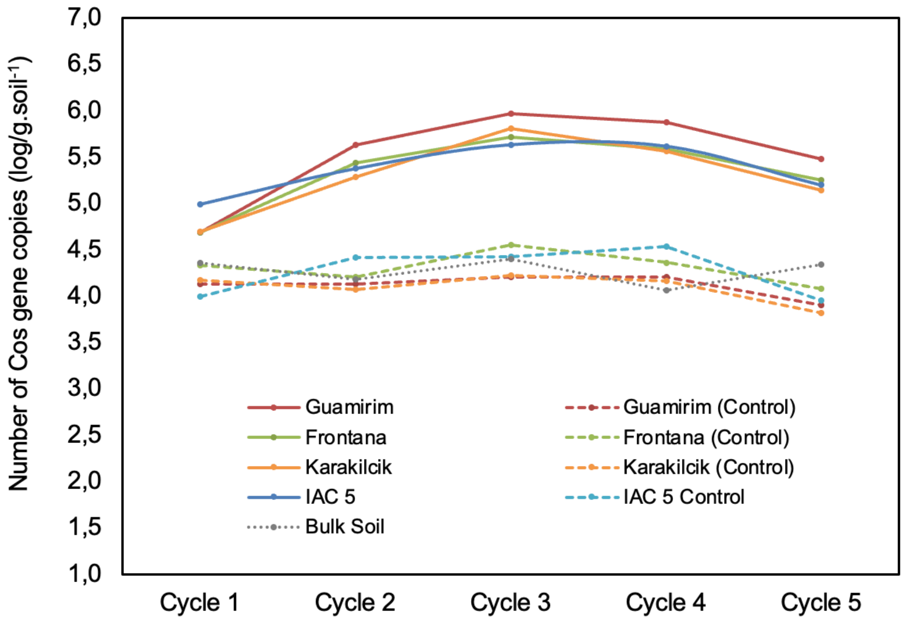

Supplementary Figure 5 Quantification of the pathogen *Bipolaris sorokiniana* in the rhizosphere and bulk soil. qPCR analysis shows the number of gene copies (log/g.soil^{-1}) present in resistant (Frontana and IAC) and susceptible (Guamirim and Karakilcik) wheat. Dashed lines are treatments without pathogen inoculation or bulk soil samples.

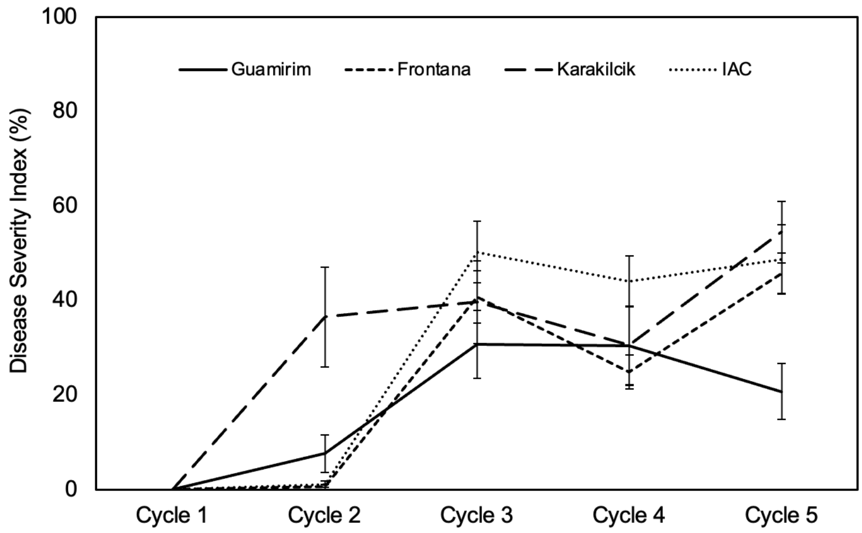

Supplementary Figure 6 Disease dynamics in resistant (Frontana and IAC 5) or susceptible (Guamirim and Karakilcik) wheat caused by indigenous pathogen, i.e. non-inoculated treatments, over five planting cycles. Bars represent the standard error of mean.

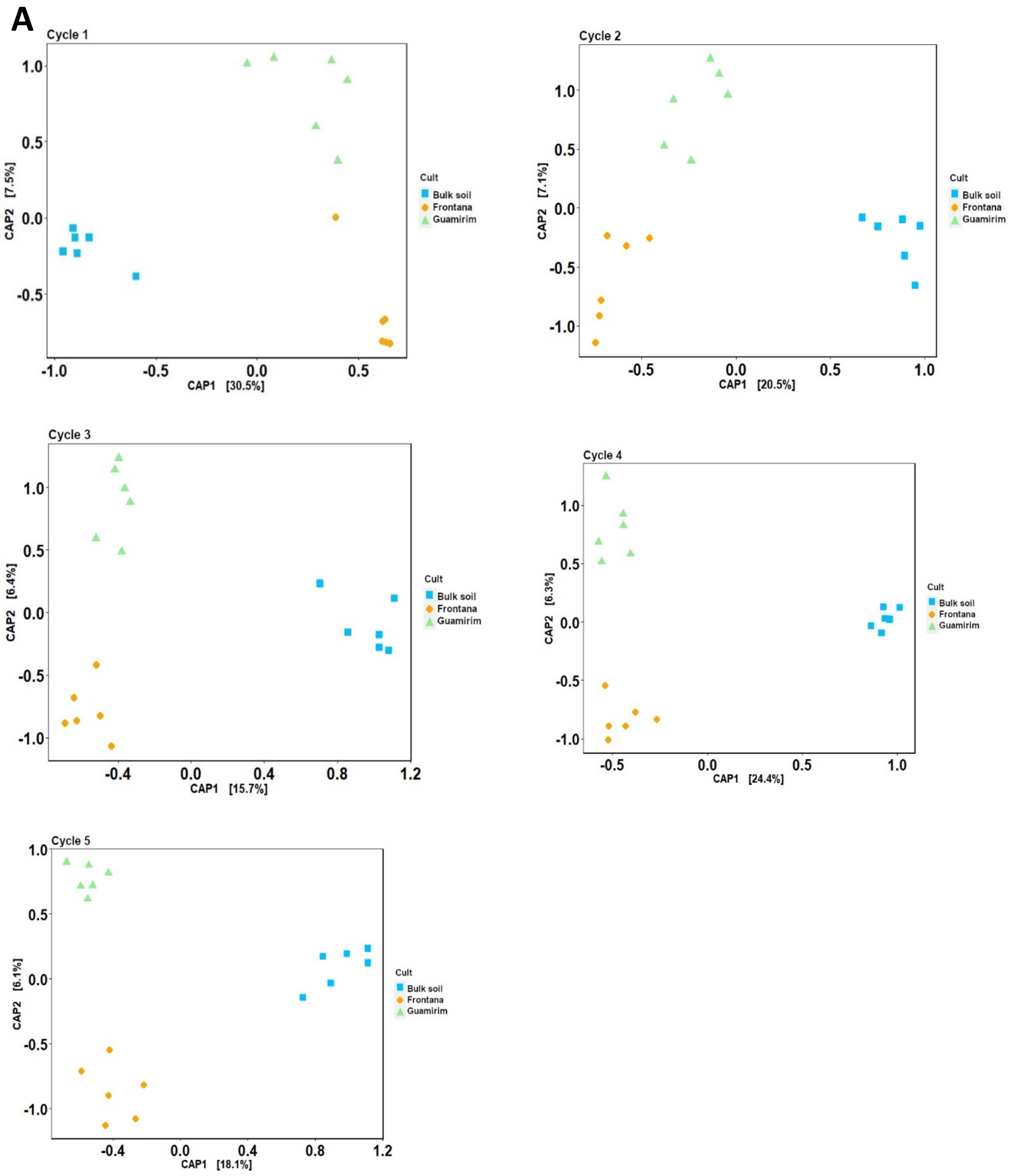

Supplementary Figure 7 (Panel B continues on the next page).

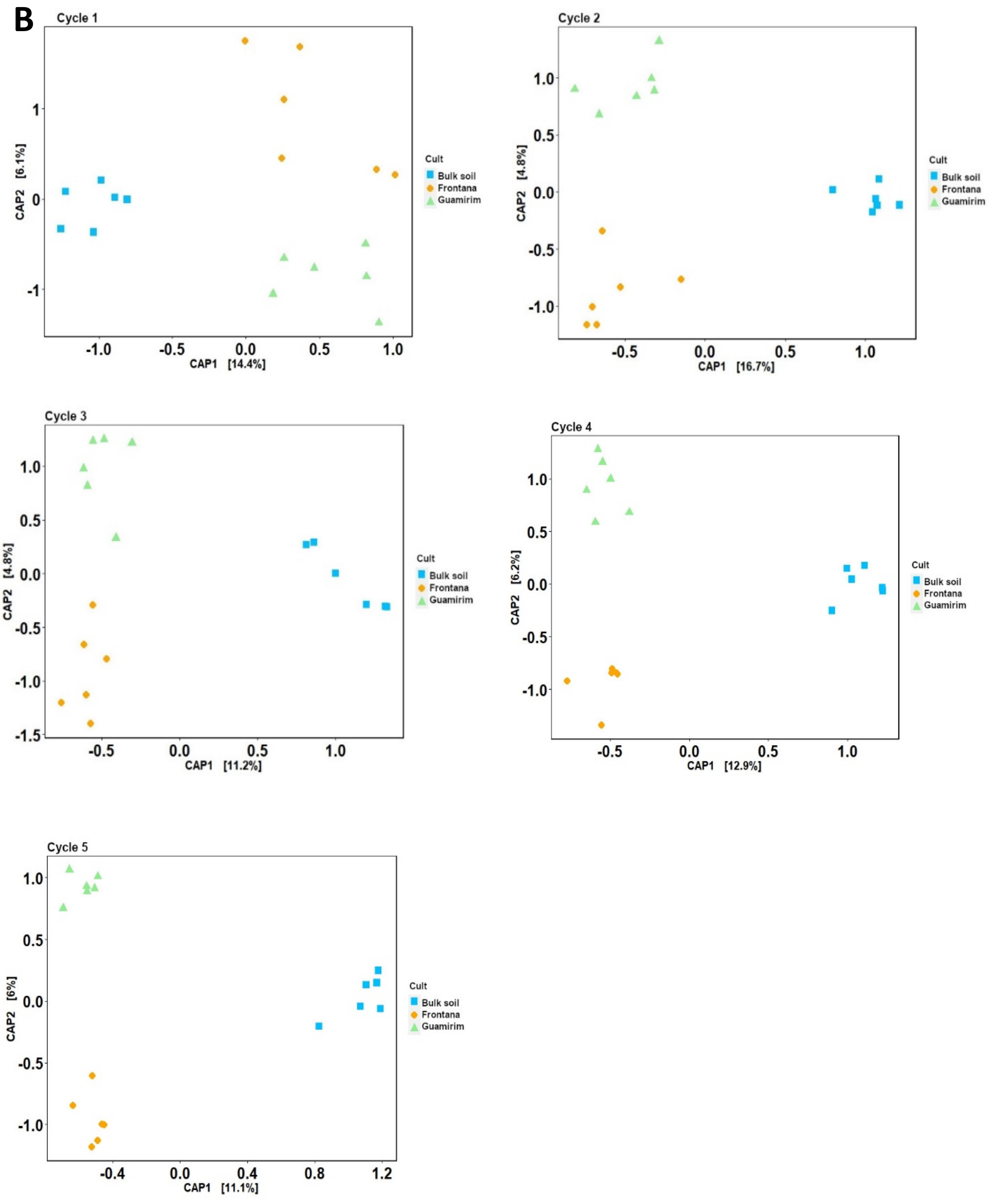

Supplementary Figure 7 Constrained Analysis of Principal Coordinates (CAP) of 16S rRNA (A) and ITS (B) amplicon beta-diversity in the rhizosphere of wheat cultivars with contrasting levels of tolerance to *Bipolaris sorokiniana*. Bacterial or fungal communities associated with the susceptible wheat Guamirim, resistant wheat Frontana, or bulk soil are shown for each one of the five growth cycles separately. Distances were calculated using Bray-Curtis Dissimilarity. Statistical significance of the constrained analysis was assessed by ANOVA, $P < 0.01$ for all presented data.

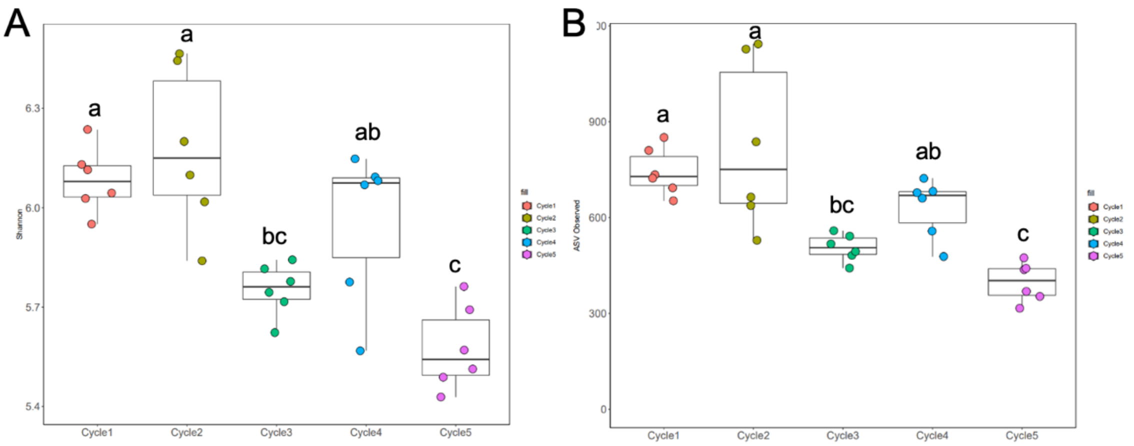

Supplementary Figure 8 Diversity based on 16S rRNA amplicons of rhizosphere microbial communities for *Bipolaris*-susceptible wheat Guamirim. A) Diversity based on Shannon's index and B) Richness based on the number of observed ASVs. Error bars represent the standard deviation of six independent replicates. Different letters indicate significant differences among treatments based on ANOVA post-hoc Tukey HSD (P < 0.05).

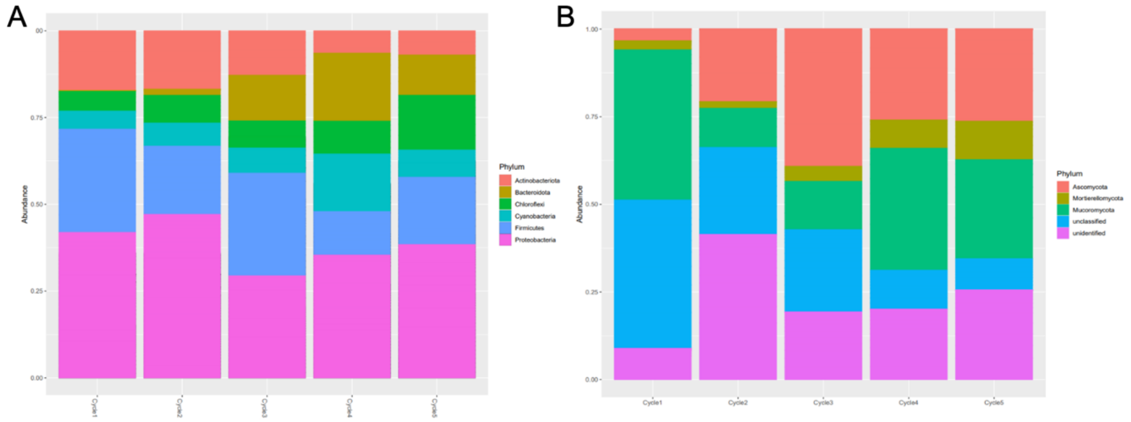

Supplementary Figure 9 Taxonomic distribution of bacterial (A) and fungal (B) phyla over five planting cycles of *Bipolaris*-susceptible wheat Guamirim. Significant differences between treatments were determined by a zero-inflated Gaussian distribution mixture model followed by moderated t-test and a Bayesian approach (FDR < 0.1).

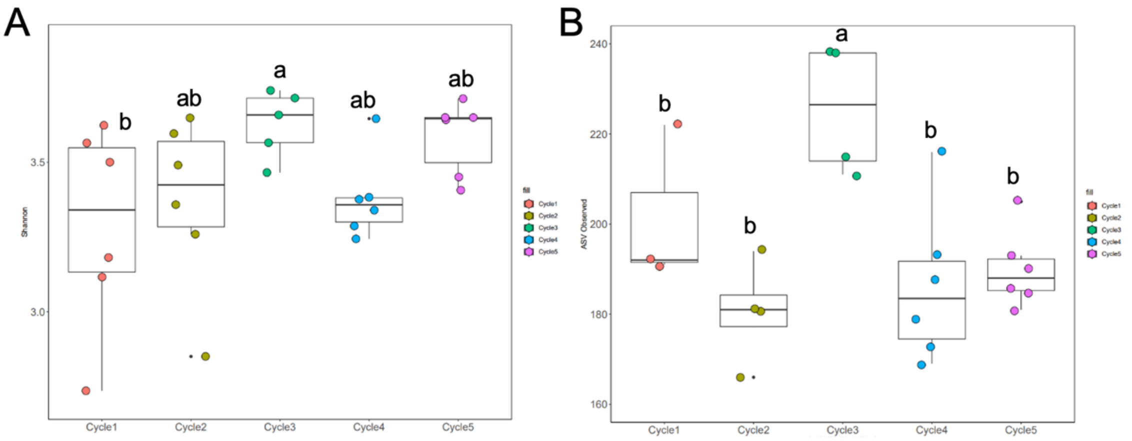

Supplementary Figure 10 Diversity based on ITS amplicons of rhizosphere microbial communities of *Bipolaris*-susceptible wheat Guamirim. A) Diversity based on Shannon's index and B) Richness based on the number of observed ASVs. Error bars represent the standard deviation of six independent replicates. Different letters indicate significant differences among treatments based on ANOVA post-hoc Tukey HSD ($P < 0.05$).

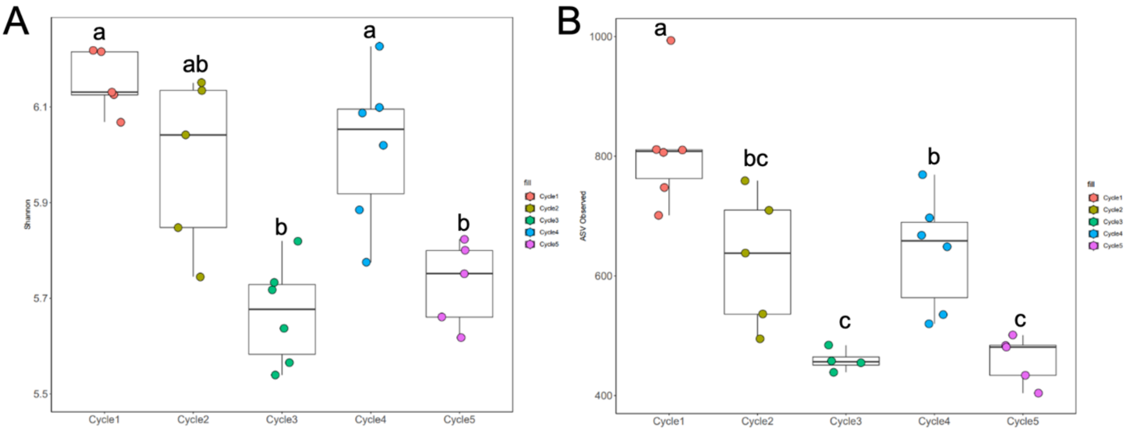

Supplementary Figure 11 Diversity based on 16S rRNA amplicons of rhizosphere microbial communities for *Bipolaris*-resistant wheat Frontana. A) Diversity based on Shannon's index and B) Richness based on the number of observed ASVs. Error bars represent the standard deviation of six independent replicates. Different letters indicate significant differences among treatments based on ANOVA post-hoc Tukey HSD ($P < 0.05$).

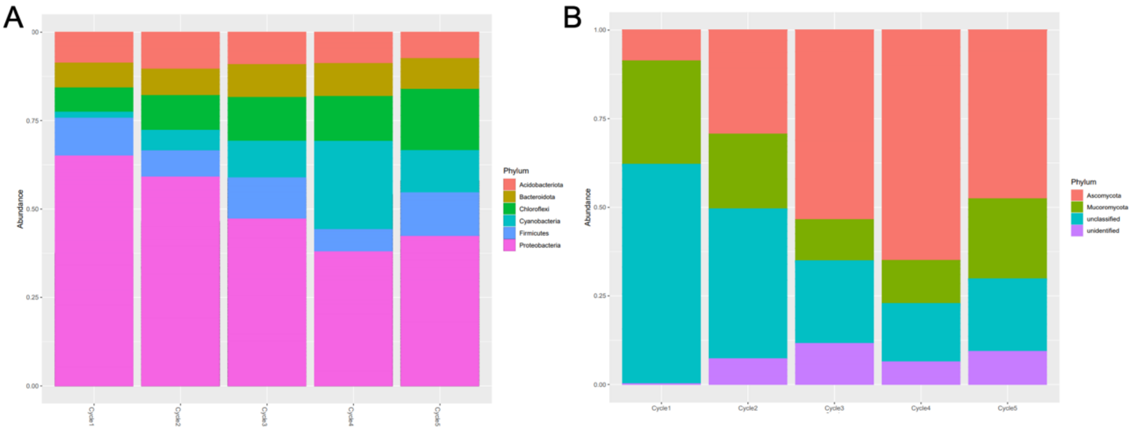

Supplementary Figure 12 Taxonomic distribution of bacterial (A) and fungal (B) phyla over five planting cycles of *Bipolaris*-resistant wheat Frontana. Significant differences between treatments were determined by a zero-inflated Gaussian distribution mixture model followed by moderated t-test and a Bayesian approach (FDR < 0.1).

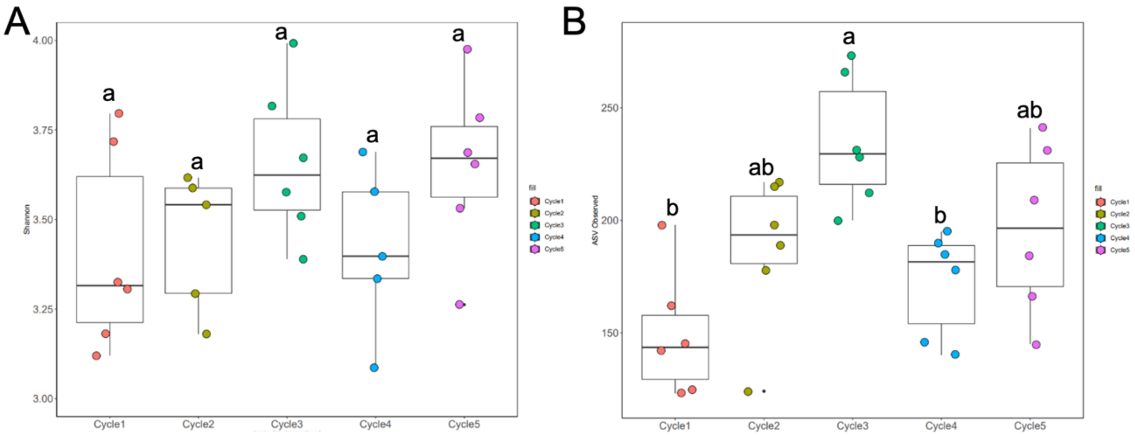

Supplementary Figure 13 Diversity based on ITS amplicons of rhizosphere microbial communities of *Bipolaris*-resistant wheat Frontana. A) Diversity based on Shannon's index and B) Richness based on the number of observed ASVs. Error bars represent the standard deviation of six independent replicates. Different letters indicate significant differences among treatments based on ANOVA post-hoc Tukey HSD (P < 0.05).

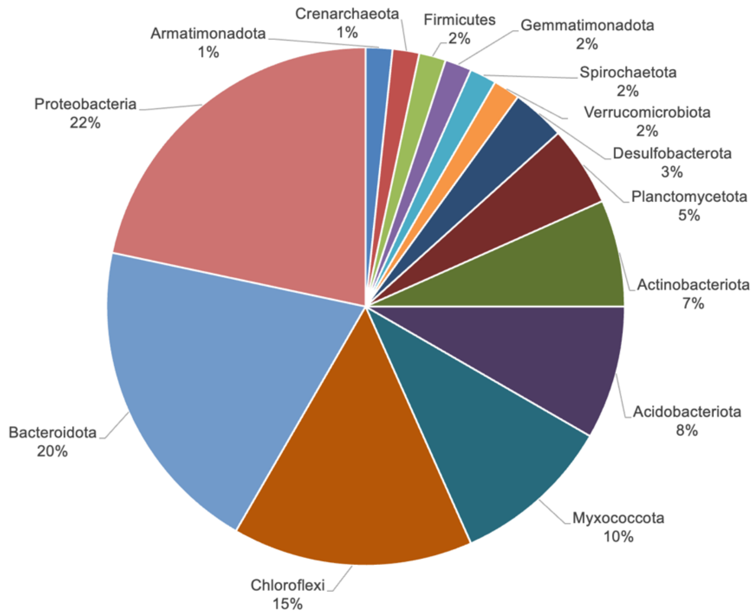

Supplementary Figure 14 Subset of bacterial phyla more abundant in cycle 4 than in cycle 1 for *Bipolaris*-susceptible wheat Guamirim (C4 > C1). Names and frequency of taxonomic groups are indicated.

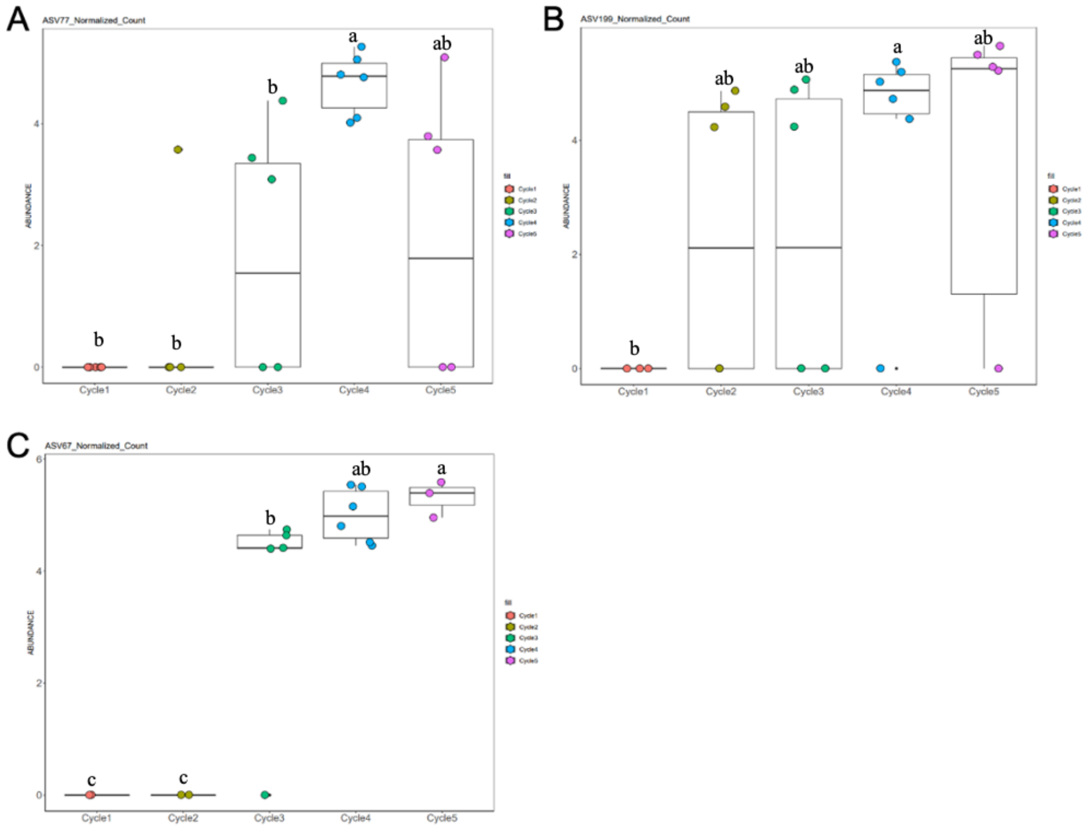

Supplementary Figure 15 Dynamics of the top 3 most dominant bacterial taxa enriched in cycle 4 correlated with low levels of disease in the susceptible wheat Guamirim. A) *Chitinophagaceae* (ASV77), B) *Nitrosomonadaceae* (ASV199) and C) *Anaerolineaceae* (ASV67). Different letters indicate significant differences among treatments based on ANOVA post-hoc Tukey HSD (P < 0.05).

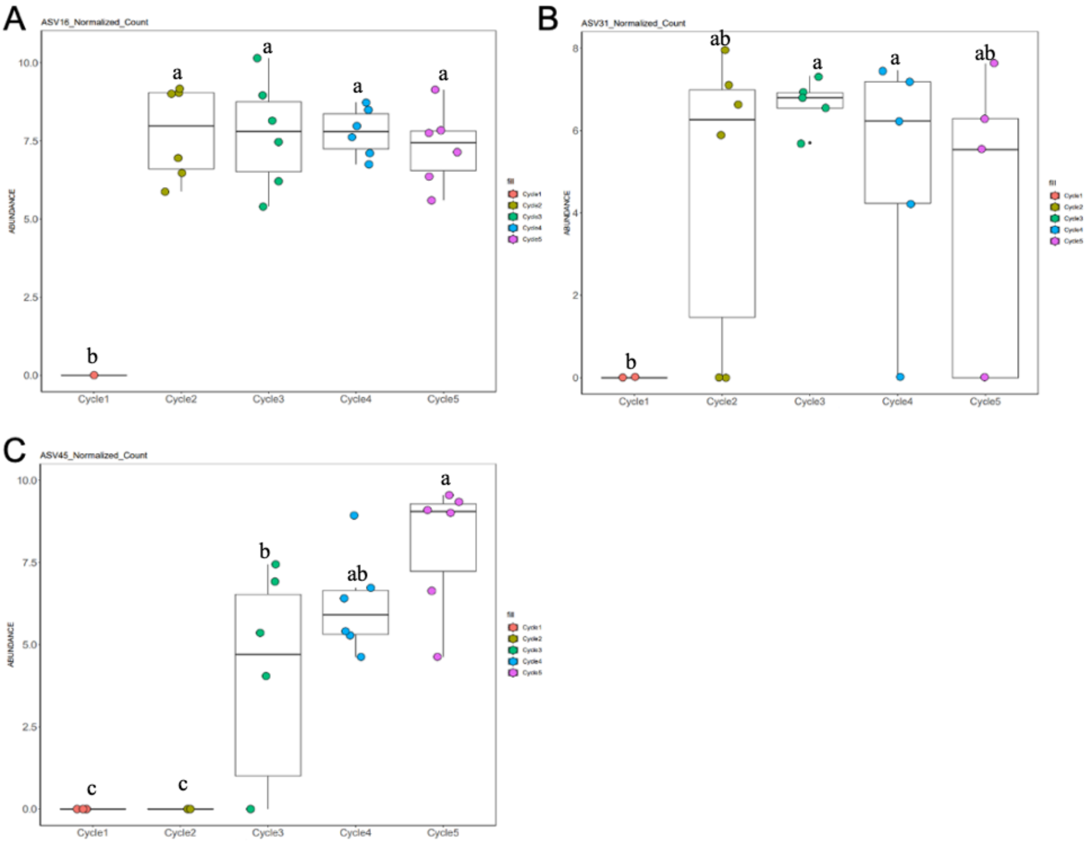

Supplementary Figure 16 Dynamics of the top 3 most dominant fungal taxa enriched in cycle 4 correlated with low levels of disease in the susceptible wheat Guamirim. A) ASV16, B) ASV31 and C) ASV45. Different letters indicate significant differences among treatments based on ANOVA post-hoc Tukey HSD ($P < 0.05$).

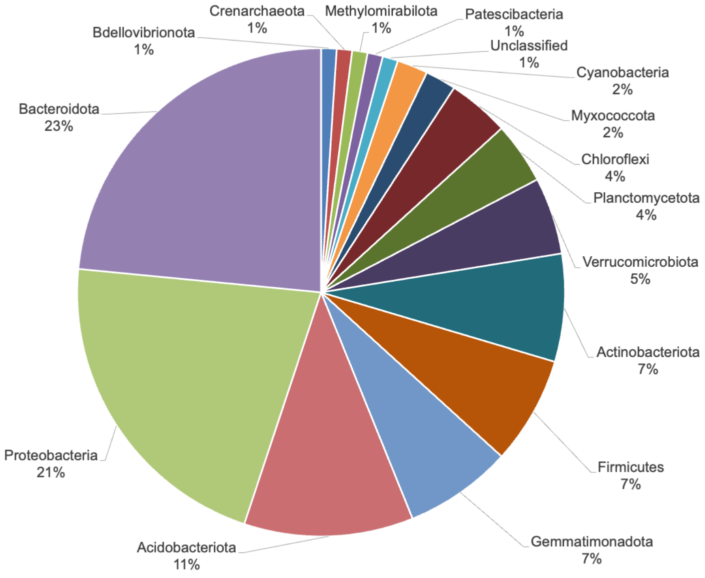

Supplementary Figure 17. Subset of bacterial phyla more abundant in cycle 1 than in cycle 4 for *Bipolaris*-resistant wheat Frontana (C1 > C4). Names and frequency of taxonomic groups are indicated.

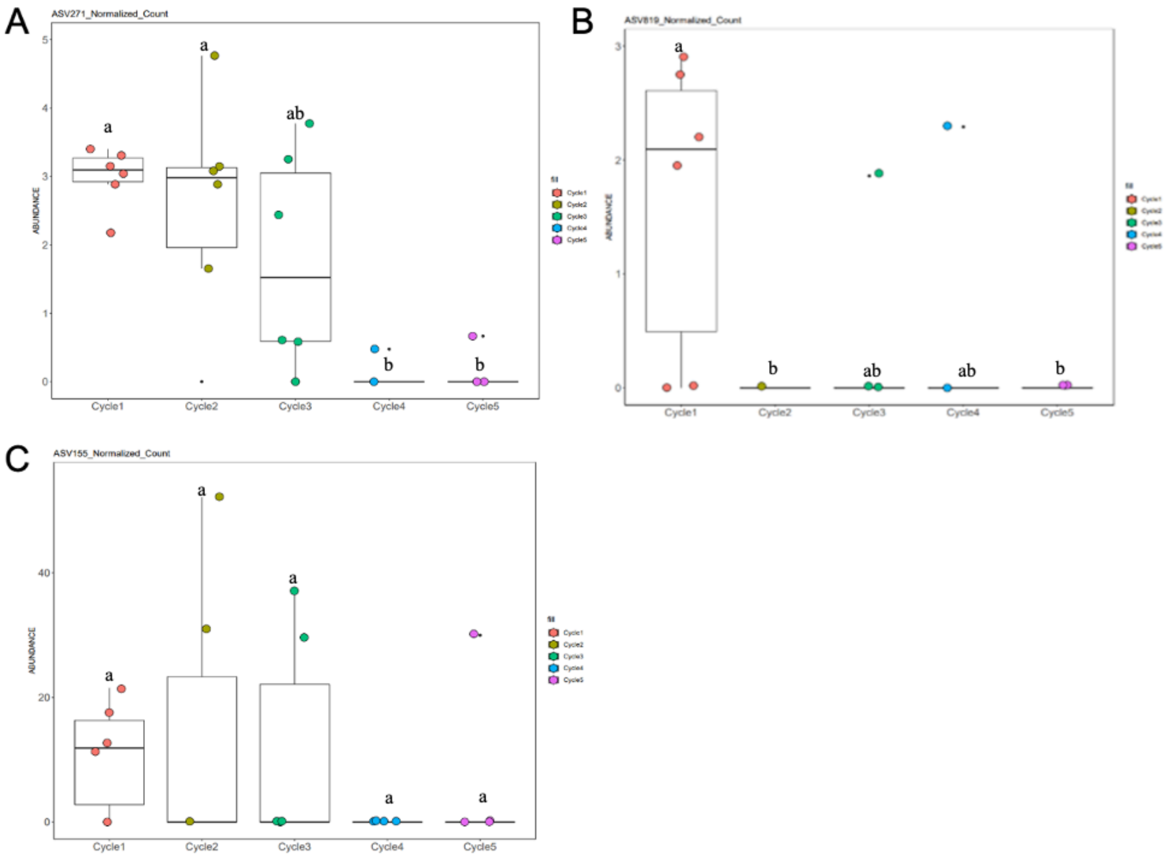

Supplementary Figure 18 Dynamics of the top 3 most dominant bacterial taxa enriched in cycle 1 correlated with low levels of disease in the resistant wheat Frontana. A) ASV271 (*Comamonadaceae*), B) ASV155 (*Bryobacteraceae*) and C) ASV819 (*Chitinophagaceae*). Different letters indicate significant differences among treatments based on ANOVA post-hoc Tukey HSD ($P < 0.05$).

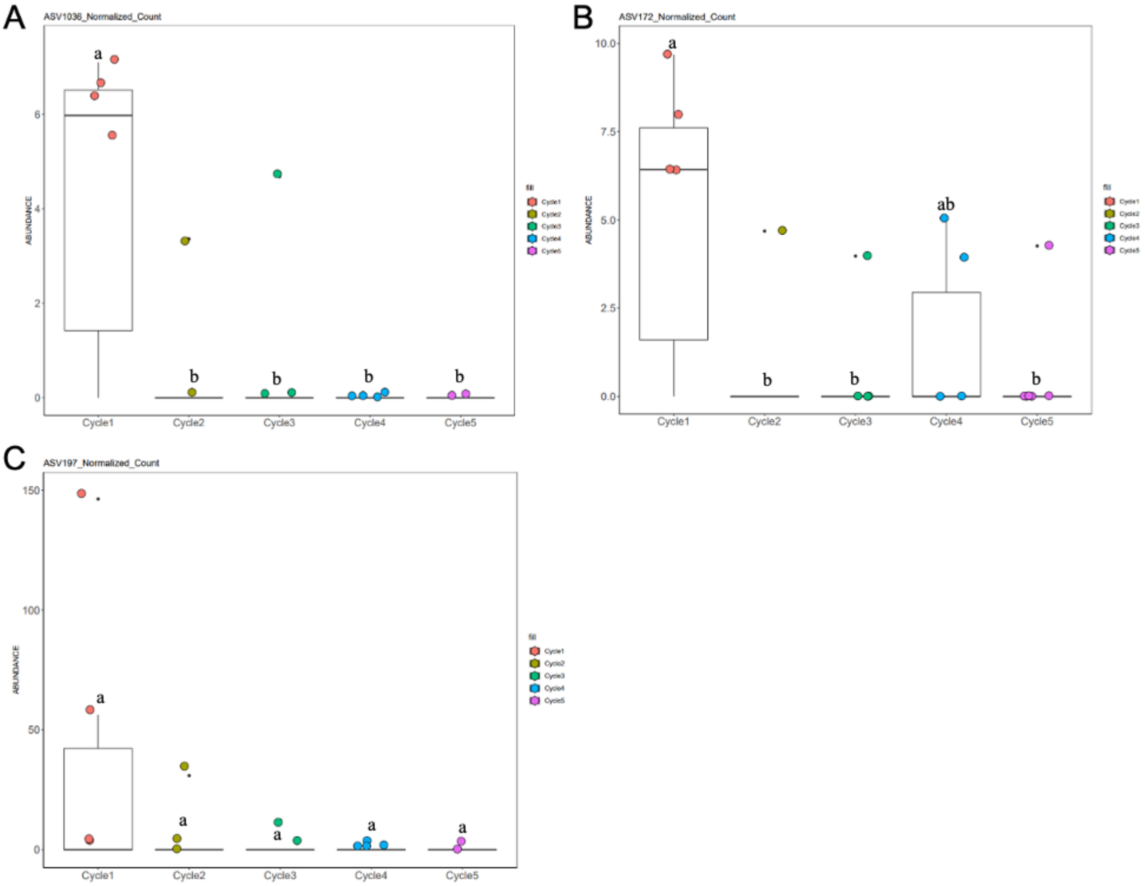

Supplementary Figure 19 Dynamics of the top 3 most dominant fungal taxa enriched in cycle 1 correlated with low levels of disease in the resistant wheat Frontana. A) ASV1036, B) ASV172 and C) ASV197. Different letters indicate significant differences among treatments based on ANOVA post-hoc Tukey HSD (P < 0.05).

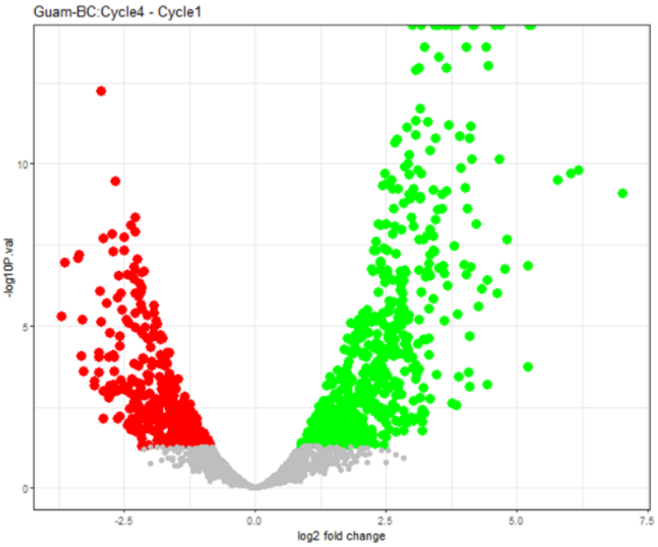

Supplementary Figure 20 Volcano plot showing differentially enriched biosynthetic gene clusters detected in the rhizosphere of the susceptible wheat Guamirim in cycle 1 and cycle 4.

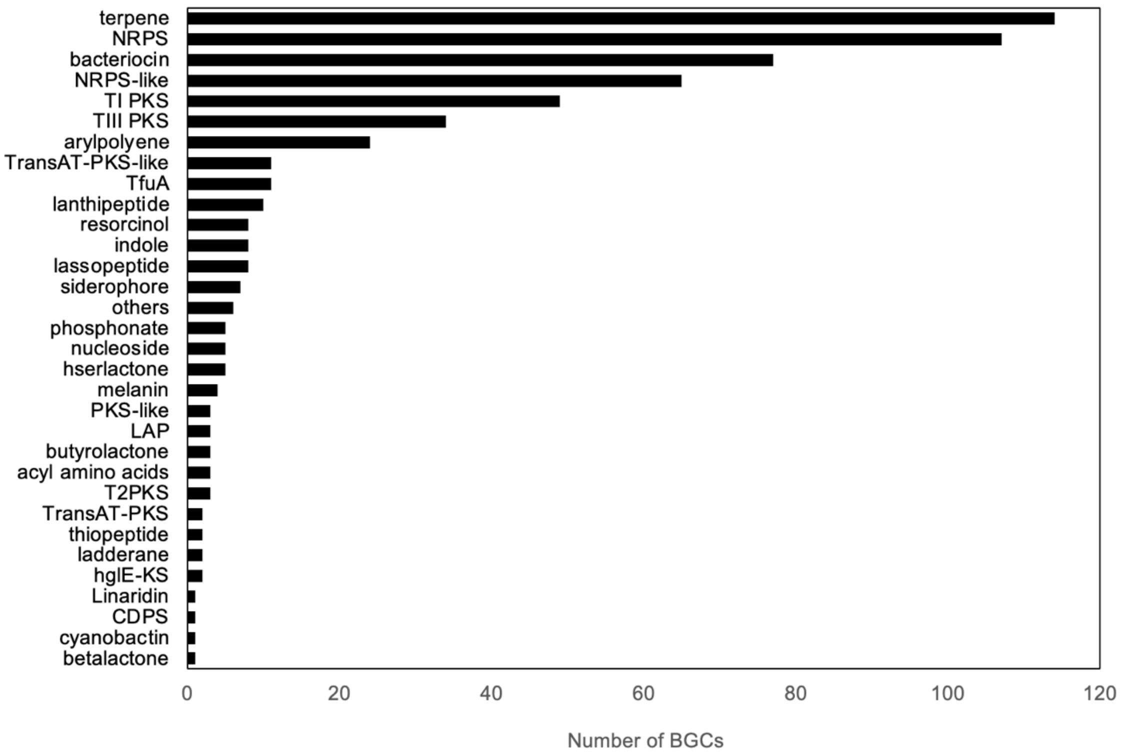

Supplementary Figure 21. Number of overrepresented biosynthetic gene clusters (BGCs) significantly enriched in cycle 4 in the rhizosphere of the susceptible wheat Guamirim.

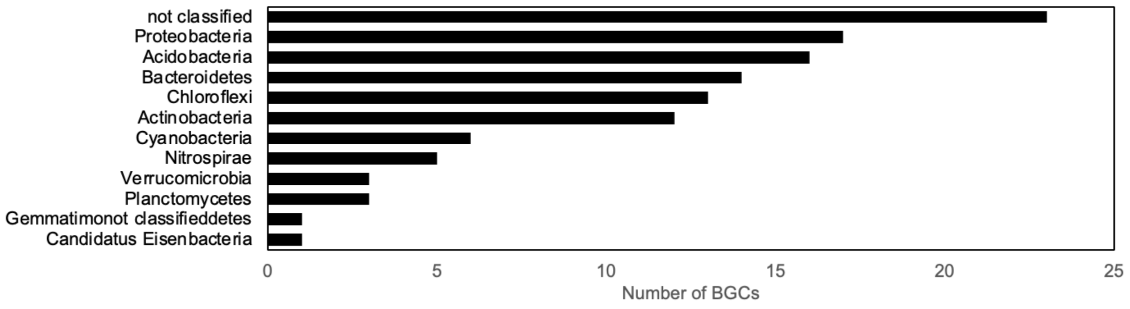

Supplementary Figure 22 Taxonomic affiliation of overrepresented terpene biosynthetic gene clusters (BGCs) detected by the antiSMASH and Clusterfinder algorithms for the different bacterial phyla in the rhizosphere of susceptible wheat Guamirim.

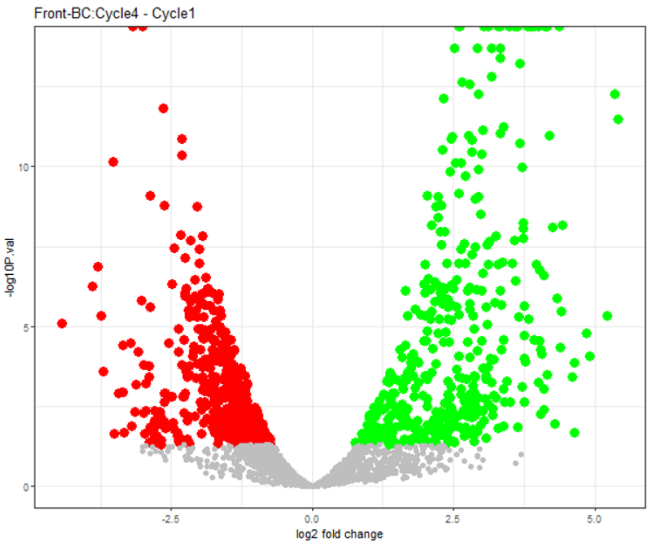

Supplementary Figure 23 Volcano plot showing differentially enriched biosynthetic gene clusters detected in the rhizosphere of the resistant wheat Frontana in cycle 1 and cycle 4.

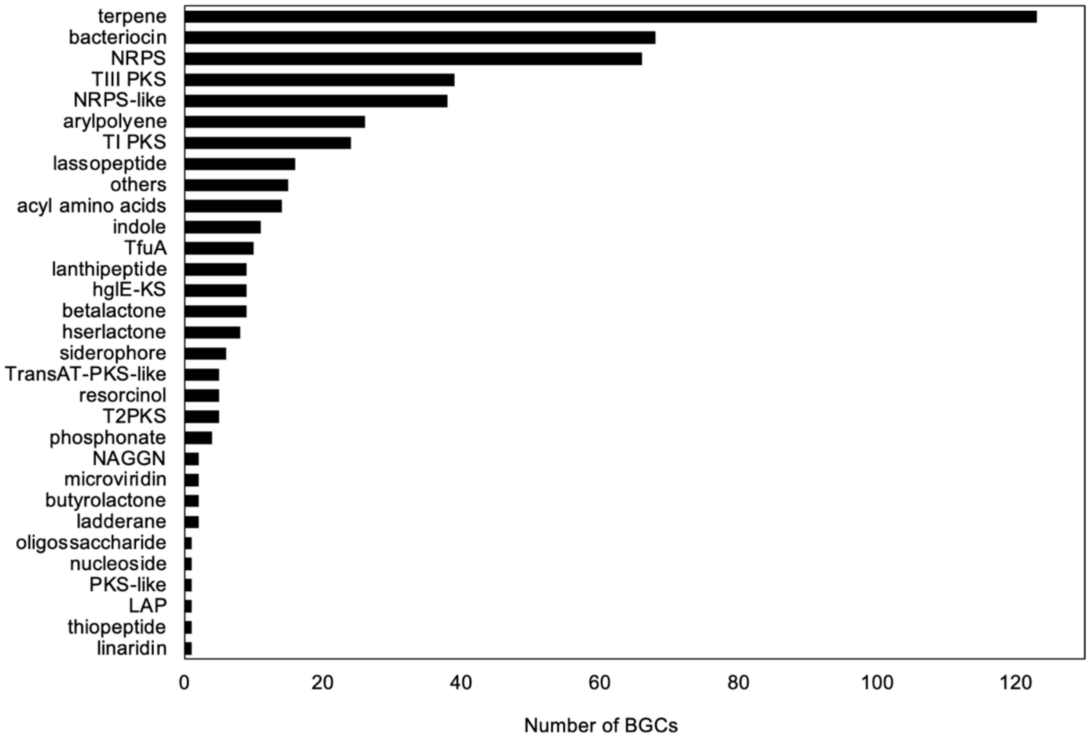

Supplementary Figure 24 Number of overrepresented biosynthetic gene clusters (BGCs) significantly enriched in cycle 1 in the rhizosphere of the resistant wheat Frontana.

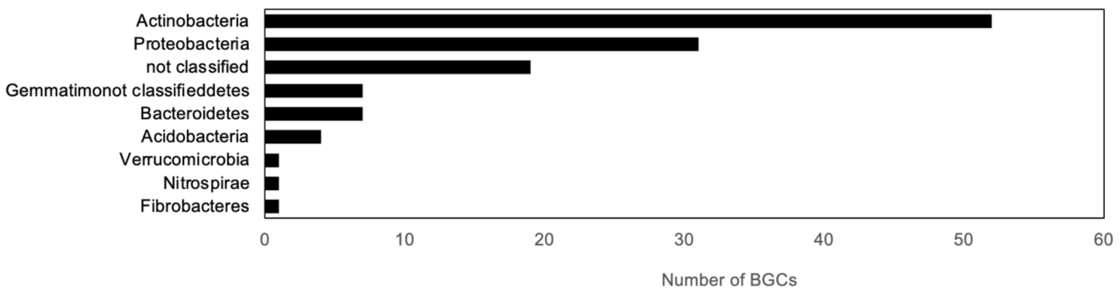

Supplementary Figure 25 Taxonomic affiliation of overrepresented terpene biosynthetic gene clusters (BGCs) detected by the antiSMASH and Clusterfinder algorithms for the different bacterial phyla in the rhizosphere of resistant wheat Frontana.
